# Overexpression of molecule GRP94 favors tumor progression in lung adenocarcinoma by interaction with regulatory T cells

**DOI:** 10.1111/1759-7714.13321

**Published:** 2020-01-22

**Authors:** Xiao‐Feng Duan, Ya‐Wei Xin

**Affiliations:** ^1^ Department of Minimally Invasive Esophageal Surgery, Key Laboratory of Cancer Prevention and Therapy, National Clinical Research Center for Cancer Tianjin Medical University Cancer Hospital and Institute Tianjin China; ^2^ The Second Hospital of Tianjin Medical University Tianjin China

**Keywords:** Bioinformatics, glucose‐regulated protein 94, lung adenocarcinoma, prognosis, Tregs

## Abstract

**Background:**

Endoplasmic reticulum stress exists within a tumor. Glucose‐regulated protein 94 (GRP94) is a stress‐induced chaperone protein involved in tumor development and progression. Its role in myeloma, colon cancer, and other tumors has been confirmed, but its role in lung cancer is unclear. This study aimed to determine the role of GRP94 in lung cancer progression and prognostic prediction.

**Methods:**

Immunohistochemical staining of GRP94 in human lung adenocarcinoma (AD) and corresponding normal tissue was performed, and its relationship with FOXP3^+^ regulatory T‐cell (Treg) infiltration analyzed. We investigated the role of GRP94 in the behavior of lung AD cells by inhibiting *GRP94* expression in A549 cells. Western blotting was used to detect the TGF‐β/SMAD2 signaling molecules and explore the possible molecular mechanism of GRP94.

**Results:**

GRP94 mRNA (encoded by *HSP90B1*) and protein levels were upregulated and elevated, respectively, in lung AD compared to normal lung tissues. High GRP94 expression was associated with an advanced disease stage and poor survival. There was a positive correlation between GRP94 expression and FOXP3^+^ Treg infiltration into lung AD tissues. Our results confirm that *GRP94* knockdown inhibits cell proliferation and promotes cell apoptosis by increasing caspase‐7 and CHOP levels in lung AD cells. TGF‐β and SMAD2 protein levels were decreased after GRP94 depletion.

**Conclusions:**

Our study revealed that that GRP94 expression in lung AD favors tumor progression and predicts poor prognosis. The oncogenic role of GRP94 may involve inducing Treg infiltration by promoting the TGF‐β signaling pathway.

**Key points:**

- GRP94 protein levels were elevated in lung AD tissues compared to normal lung tissues. The high expression of *GRP94* in lung AD favors tumor progression and predicts poor prognosis.
- The oncogenic role of the molecule GRP94 may involve the stimulation of Treg infiltration *via* promotion of the TGF‐β signaling pathway.

## Introduction

Lung cancer is the most common malignancy worldwide.^1^, ^2^ The majority of lung cancers are non‐small cell lung cancers (NSCLC), of which adenocarcinoma (AD) is the most common pathological type. Although there has been great progress in terms of early diagnosis and comprehensive therapy, the five‐year overall survival rate of NSCLC is still less than 20%.^3^ Hence, it is necessary to study the pathogenesis of lung cancer. A wide range of stress conditions may exist within a tumor, including hypoxia, changes in redox homeostasis, altered cell metabolism, acidosis, fast cell proliferation, and increased protein production and synthesis, all of which can trigger endoplasmic reticulum (ER) stress.^4^ Glucose‐regulated protein 94 (GRP94) is a stress‐inducible molecular chaperone belonging to the heat shock protein (HSP) 90 family.^5^ GRP94 is upregulated in response to many stress conditions and plays a key role in regulating the balance between cancer cell survival and apoptosis by maintaining the ability of ER protein folding. In addition, GRP94 is responsible for the chaperoning of several essential proteins, such as TLRs (except TLR3),^6^ Wnt co‐receptor LRP6,^7^ GARP,^8^ and insulin‐like growth factor,^9^ as well as the majority of α‐ and β‐integrin subunits.^10^, ^11^ These GRP94 client proteins play roles in different stages of cancer development, indicating that GRP94 has a key role in tumorigenesis.^12^ Previous studies have suggested that GRP94 promotes tumorigenesis in many tumor models, including multiple myeloma,^13^ colitis‐associated colon tumorigenesis in mice,^14^, ^15^ and liver cancer.^16^, ^17^, ^18^ However, studies of GRP94 function in lung cancer are limited. The purpose of this study was to determine the role of GRP94 in lung cancer progression and prognostic prediction.

## Methods

### Patients

This study included 80 patients with lung AD who were admitted to Tianjin Medical University Cancer Research Institute and Hospital between 2012 and 2014. The Cancer Research Institute of Tianjin Medical University and the hospital ethics committee approved the use of patient samples and information.

### Bioinformatic analysis

Through an Oncomine Research Premium edition upgrade, downloaded raw datasets that included mRNA expression, clinical and pathological information, and survival data (Thermo Fisher, Ann Arbor, MI; http://www.oncomine.org). A Kaplan‐Meier plotter (http://www.kmplot.com) was used to confirm the prognostic significance of the HSP90B1 mRNA expression in lung cancers.^19^ The desired Affymetrix ID of the *HSP90B1* gene was 200599_s_at.

### Cell culture and treatments

Human lung AD A549 cells were preserved in our hospital. The A549 cells were cultured in an RPMI 1640 medium supplemented with a 1% penicillin/streptomycin mixture and 10% fetal bovine serum (Bio Industry, Israel). The cells were stored in a humidified incubator containing 5% CO_2_ at 37°C.

### Antibodies and reagents

The antibodies (CHOP, caspase‐7, TGF‐β, and SMAD2) used for western blotting were provided by Cell Signaling Technology (Danvers, MA, USA). The GRP94 (9G10) and FOXP3 antibodies were purchased from Santa Cruz Biotechnology, Inc. A DBA substrate ABC kit was obtained from Vector Laboratorie. All other chemicals were obtained from Sigma‐Aldrich and Fisher Scientific. The small interfering RNA (siRNA) for GRP94 was provided by Ribobio (Guangzhou, China).

### Immunohistochemical staining

Specimens were processed using formalin fixation, embedded in paraffin, and sectioned to a thickness of 5 μm. Immunohistochemistry (IHC) for GRP94 and FOXP3 was performed manually. Tissue sections were placed at 60°C for two hours and hydrated with xylene and gradient alcohol. Antigen reparation was performed by steaming samples in a sodium citrate buffer at 95°C for 20 minutes. The slides were then permeabilized with cold methanol at −20°C for five minutes. Endogenous peroxidase activity was quenched by incubating with a peroxidase block. Slides were blocked with 2% BSA and 10% NGS in PBS at room temperature (RT) for two hours. The slides were incubated in 1% BSA, 1% NGS/PBS with a rat anti‐human GRP94 antibody (1:200 dilution) and a rabbit anti‐human FOXP3 antibody (1:150 dilution) for one hour at RT. Biotin anti‐rat and biotin anti‐rabbit IgG were applied to the slides for 30 minutes (ABC kit, Vector Laboratories, Inc) at RT, and signal detection was performed using 3,3′‐diaminobenzidine tetrahydrochloride (DAB Kit, Vector Laboratories, Inc). Slides were counterstained with hematoxylin, dehydrated, and mounted. The slides were reviewed and scored blindly by an experienced pathologist. The staining intensity was scored as follows: 0, negative staining; one, weak staining; two, moderate staining; three, strong staining; and four, very strong staining. Images of the slides were taken at 100× and 400× magnification with a universal vertical fluorescence microscope and imaging system (Olympus BX61, Japan). Tregs were counted as the number of FOXP3^+^ lymph cells in the lung cancer microenvironment (high Treg group ≥10 Treg cells/high‐power field [HPF]; low Treg group <10 Treg cells/HPF).

### siRNA interference

Cells were plated into six‐ or 24‐well plates and transfected with a GRP94 siRNA and a negative control siRNA. According to the manufacturer's instructions, the lung cancer cell line, A549, was transfected using Lipofectamine 3000 (Invitrogen, Carlsbad, CA, USA). Western blotting was used to detect the transfection efficiency.

### Cell viability assay

According to the manufacturer's instructions, cell viability was assessed by a cell count kit‐8 (CCK‐8) (Dojindo, Kumamoto, Japan). A549 cells were seeded on a 96‐well plate and left to grow for two days. The optical density was measured at 450 nm.

### Cell apoptosis analysis

According to the manufacturer's instructions, apoptosis was evaluated with an Annexin V‐FITC apoptosis detection kit (Thermo Fisher Waltham, MA, USA). In brief, A549 cells were collected, washed with a binding buffer, and stained with Annexin V‐FITC and propidium iodide before quantification via flow cytometry.

### Western blotting

A sodium dodecyl sulfate (SDS) lysate buffer was used to lyse the total protein of cultured cells. The protein concentration was determined using a BCA analysis kit (Thermo Fisher Scientific, USA). Based on whether 8% or 10% SDS polyacrylamide gel electrophoresis was used, the same amount of protein was isolated and transferred to a polyvinylidene difluoride (PVDF) membrane. Reactions with GRP94, caspase‐7, CHOP, TGF‐β, SMAD2, and β‐actin were then performed overnight at 4°C. The PVDF membrane was then washed with PBS‐Tween (PBST) buffer and incubated with Goat anti‐mouse/rabbit IgG (H + L)‐horseradish peroxidase (HRP) (ray antibody biotechnology company, Beijing, China) for one hour. The bands were visualized using an electrochemiluminescence liquid (Merk, Germany), and images were taken using a Tanon 6600 luminous imaging workstation (Tanon, China).

### Statistical analysis

SPSS version 22.0 (SPSS Inc., Chicago, IL, USA) and GraphPad Prism software (La Jolla, CA, USA) were used for all statistical analyses. An unpaired two‐tailed Student's *t*‐test or a Mann‐Whitney test was used to compare the differences between groups of nonparametric data, and a Chi‐square test or a Fisher exact test was used to compare differences between groups of parametric data. Survival curves were plotted using the Kaplan‐Meier method and compared using a log‐rank test. The receiver operating characteristics (ROC) curve was used to determine the optimal cutoff point of gene expression in low‐ and high‐risk patients. A two‐sided *P*‐value of less than 0.05 was considered statistically significant (*P < 0.05; **P < 0.01; ***P < 0.001; ****P < 0.0001).

## Results

### Expression of the *GRP94*‐encoding gene is elevated in lung adenocarcinoma

**Figure 1:**
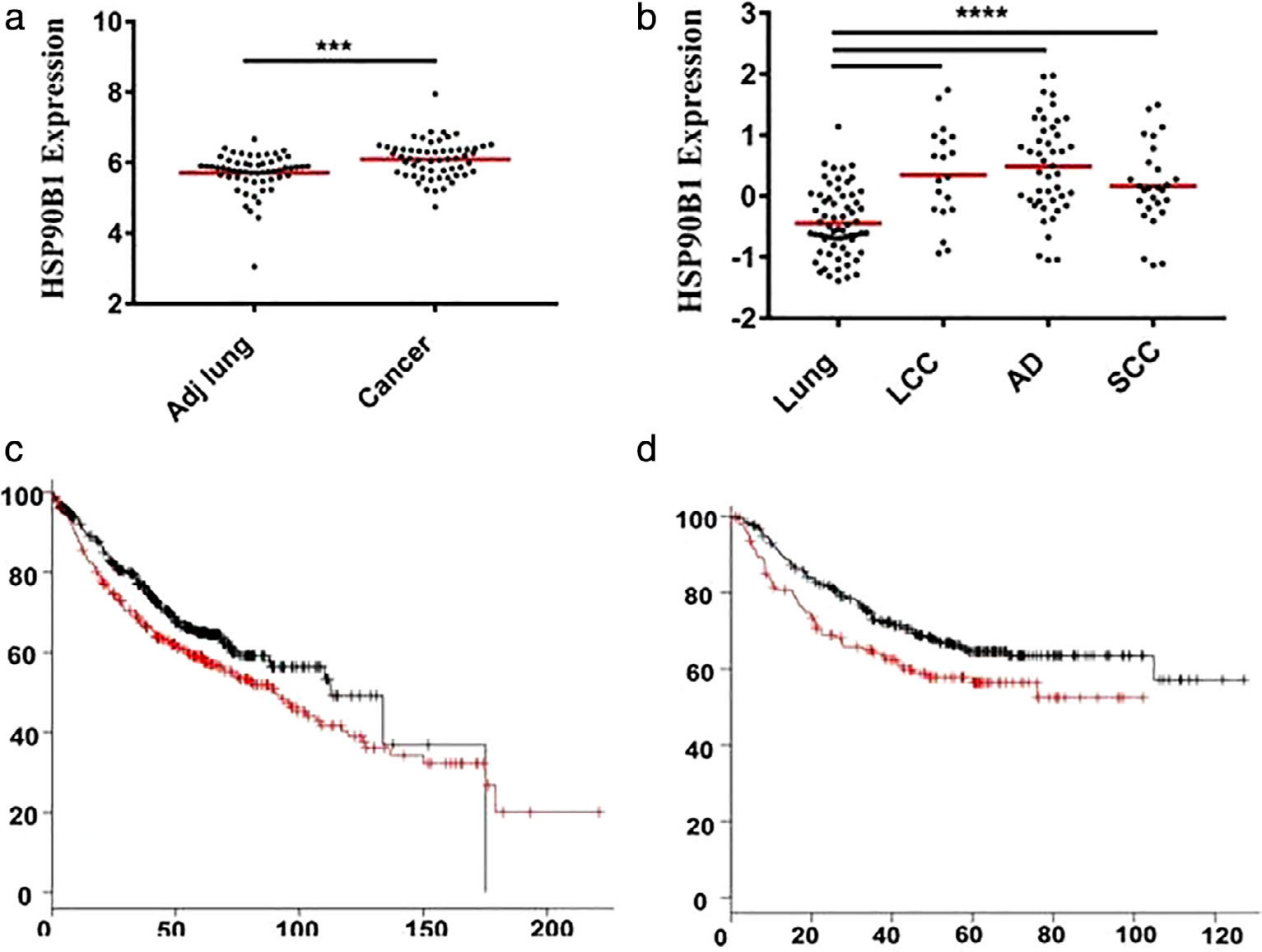
*HSP90B1* expression in lung cancer tissue and prognostic value. (**a**) *HSP90B1* was upregulated in lung cancer tissue compared with normal lung tissue in the Landi dataset (*P* < 0.0001). (**b**) *HSP90B1* was upregulated in lung large cell carcinoma (LCC), squamous cell carcinoma (SCC) and adenocarcinoma (AD) tissue compared with normal lung tissue in the Hou dataset, respectively (*P* < 0.001). (**c**) The Kmplot database was used to confirm the predictive value of HSP90B1 in lung AD, and a high level of *HSP90B1* expression was significantly correlated with a worse overall survival [HR = 1.3 (1.03–1.64), *P* = 0.029] (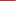) Hi and (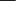) Lo. (**d**) A high level of *HSP90B1* expression was significantly correlated with a worse progression‐free survival in lung AD [HR = 1.45 (1.05–2)，*P* = 0.024] (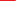) Hi and (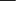) Lo.

**Figure 2:**
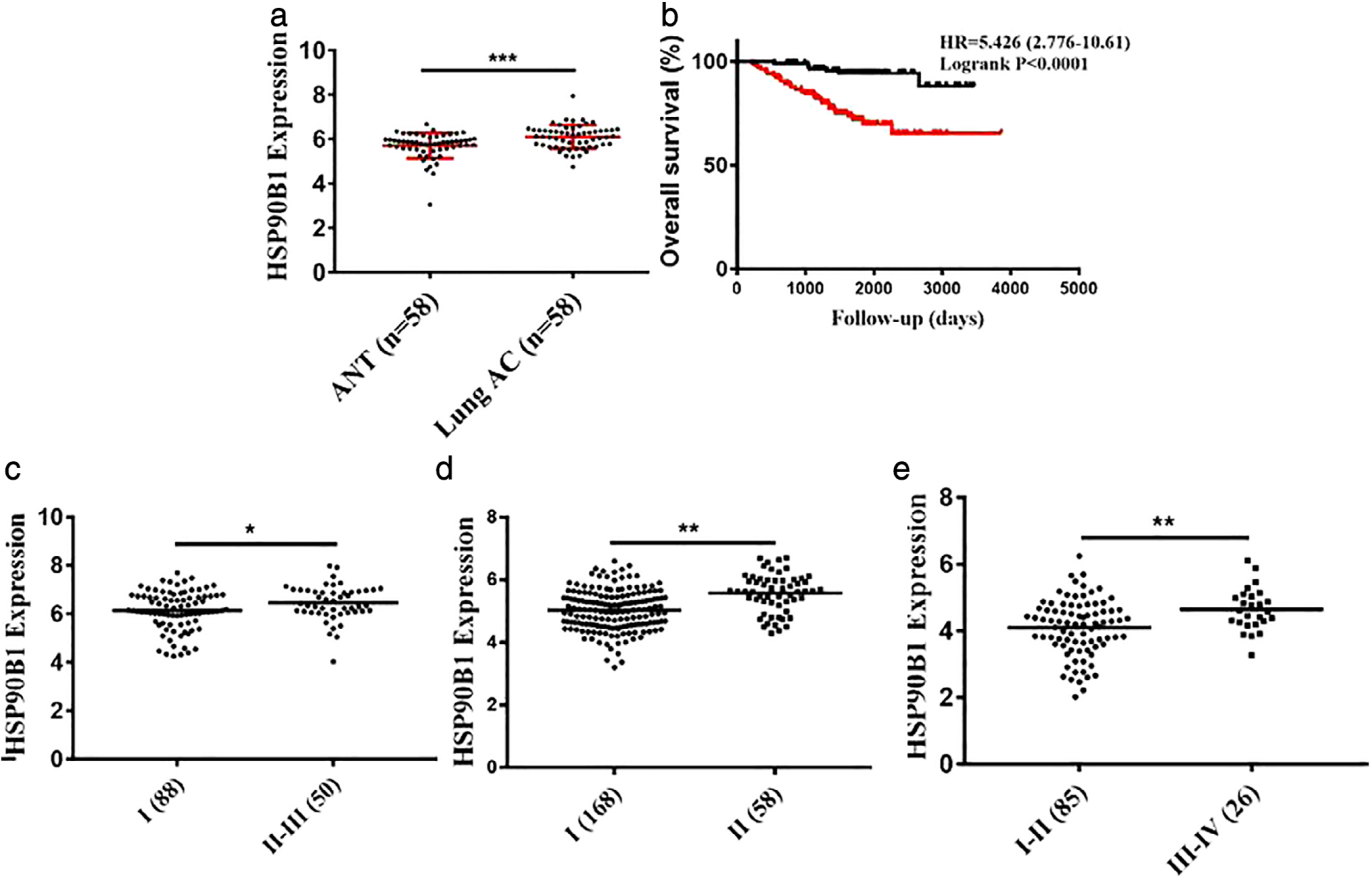
*HSP90B1* expression in lung AD tissue and clinical value. (**a**) *HSP90B1* was upregulated in lung AD tissue compared with normal lung tissue in the Selamat dataset (*P* < 0.001). (**b**) By analyzing the Okayama lung dataset, patients with a higher level of *HSP90B1* expression had a worse rate of overall survival (*P* < 0.0001) (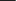) Low expression and (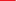) High expression. (**c**) Lung AD with an advanced disease stage had higher *HSP90B1* expression (*P* < 0.05). (**d**) Lung AD with an advanced disease stage had higher *HSP90B1* expression (*P* < 0.01). (**e**) Lung AD with an advanced disease stage had higher *HSP90B1* expression (*P* < 0.01).

The raw data, including *HSP90B1* (*GRP94*‐encoding gene) expression, clinical and pathological information, and survival status, were downloaded from an Oncomine Premium Edition upgrade and analyzed (detailed information of the Oncomine cohort can be found in the Supporting information). We first investigated *HSP90B1* expression in human lung cancer. Of the 13 datasets analyzed, all of which compared *HSP90B1* expression in lung cancer and normal lung tissue, 10 studies consistently showed that *HSP90B1* was upregulated in lung cancer tissue, one study indicated that *HSP90B1* was downregulated in lung carcinoid tumors, and two studies showed no differential gene expression (Table 1). Representatively, *HSP90B1* was upregulated in lung cancer tissue compared with normal lung tissue in the Landi (Fig 1a, *P* < 0.0001), Hou (Fig 1b, *P* < 0.001), and Selamat datasets (Fig 2a, *P* < 0.001).

**Table 1:** *HSP90B1* differential expression in lung cancer compared with lung tissue

	HSP90B1 gene expression		
Dataset	Lung tissue, n	Lung cancer, n	*P*‐value
Landi	49	AD†, 58	<0.0001
Okayama	20	AD, 226	<0.01
Selamat	58	AD, 58	<0.001
Wei	25	AD, 25	<0.01
Stearman	19	AD, 20	<0.01
Su	30	AD, 31	<0.01
Talbot	2	SCC‡, 34	<0.05
Wachi	5	SCC, 5	<0.05
Yamagata	3	LCC§, 5	<0.01
		AC, 9	<0.05
		SCC, 11	<0.05
Hou	65	LCC, 19	<0.001
		AD, 45	<0.001
		SCC, 17	<0.001
Hhattacharjee	17	Carcinoid tumor, 20	<0.0001¶
Garber	5	AD, 40	>0.05
		LCC, 4	>0.05
		SCC, 13	>0.05
		Small cell cancer, 4	>0.05
Beer	10	AD, 86	>0.05

† Adenocarcinoma.

‡ Squamous cell carcinoma.

§ Large cell carcinoma.

¶ Underexpression in carcinoid tumor.

### Elevated *HSP90B1* expression correlates with a poor clinical outcome

We next examined whether *HSP90B1* expression in lung cancer cells has any clinical significance by analyzing the raw data from the Oncomine Premium Edition upgrade. Three datasets showed that tumors with an advanced disease stage had higher *HSP90B1* expression (Fig 2c, *P* < 0.05; Fig. 2d, *P* < 0.01; Fig 2e, *P* < 0.01). We next determined whether the *HSP90B1* expression level could predict survival. By analyzing the Okayama lung dataset, we identified that patients with a higher level of *HSP90B1* expression had a worse rate of overall survival (Fig 2b, *P* < 0.0001). Furthermore, the Kmplot database was also used to confirm a consistent predicting value for the clinical outcome. We found that a high level of *HSP90B1* expression was significantly correlated with a worse overall survival and progression‐free survival in lung AD patients (Fig 1c, *P* = 0.029; Fig 1d, *P* = 0.024), but not in lung SCC patients (data not shown).

### GRP94 protein levels are increased in lung adenocarcinoma

**Figure 3:**
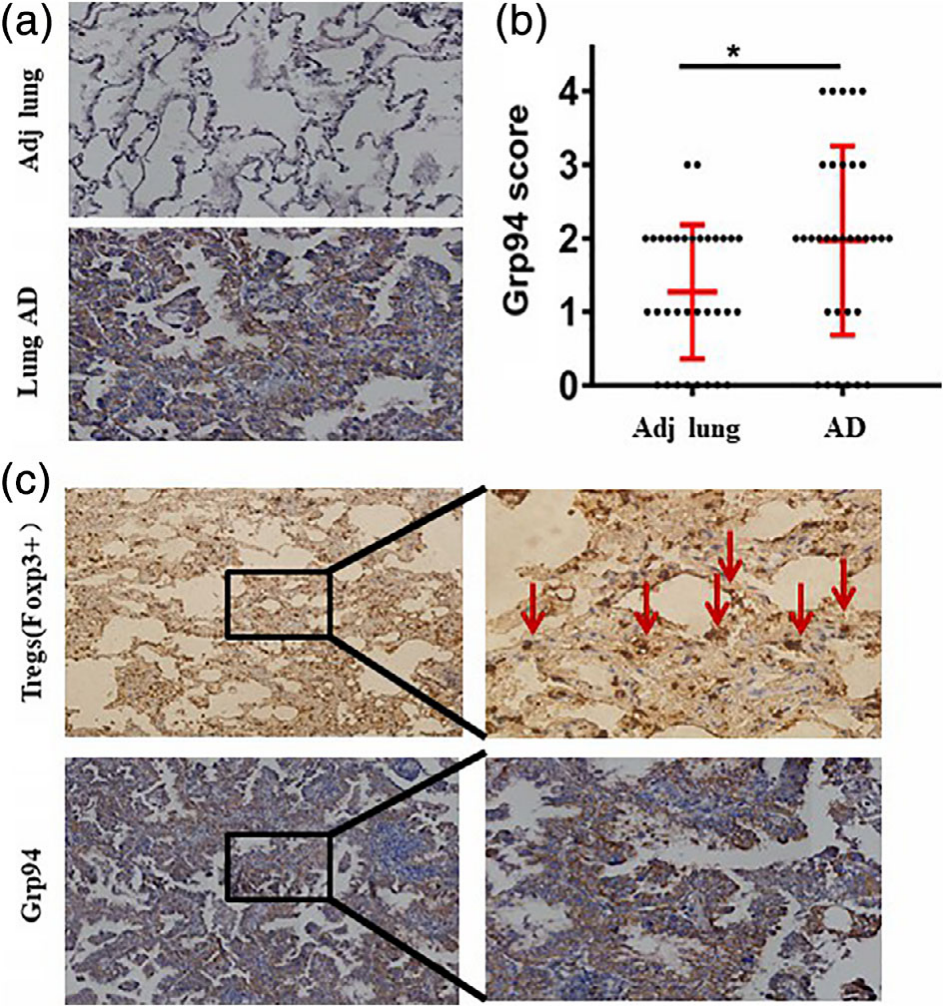
GPR94 expression in lung AD and the interaction with FOXP3. (**a**) GRP94 protein levels in 33 lung AD tissue and adjacent normal lung tissue (ANT) samples by IHC. (**b**) GRP94 was overexpressed in lung AD tissue compared with ANT samples (*P* < 0.05). (**c**) The expression level of FOXP3 in lung AD tissue indicated FOXP3^+^ Tregs infiltrated into the tumor microenvironment.

We examined GRP94 protein levels in 33 lung AD tissue and adjacent normal lung tissue (ANT) samples by IHC (Fig 3a). This revealed that GRP94 was overexpressed in lung AD tissue compared with ANT samples (Fig 3b, *P* < 0.05).

### Overexpression of GRP94 correlates with an aggressive clinical outcome

**Figure 4:**
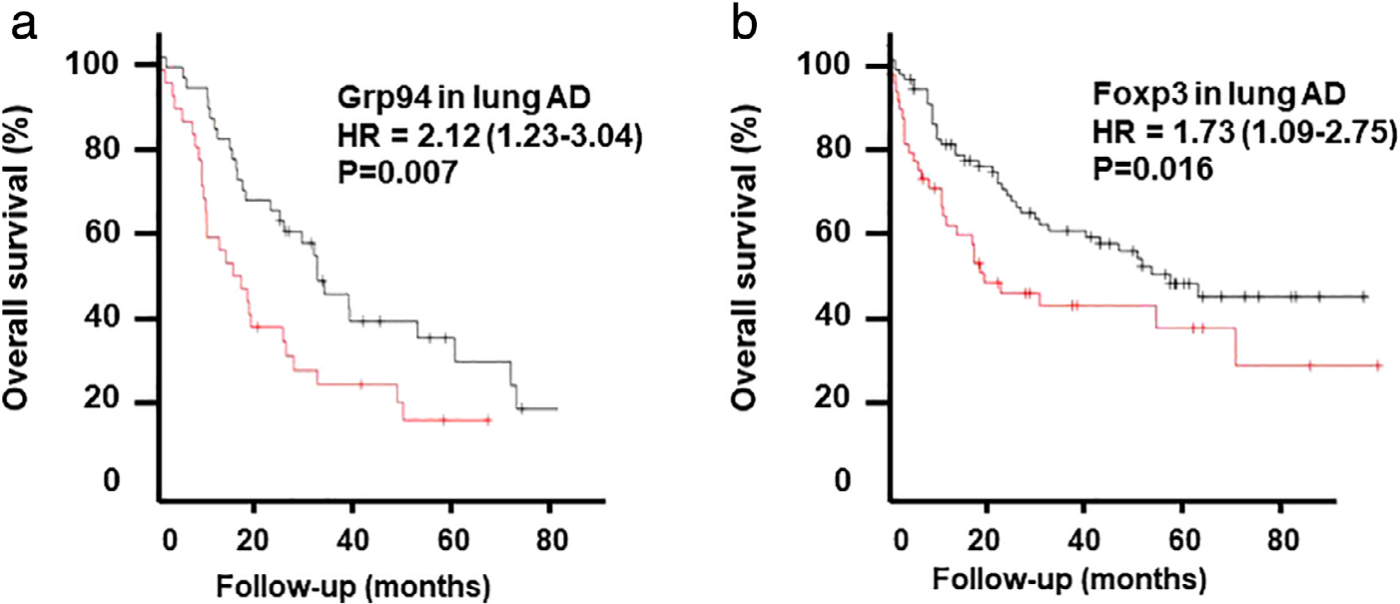
GPR94 expression in lung AD predicts overall survival. (**a**) Lung AD patients with high levels of GRP94 had a significantly shorter overall survival than those with low levels of GRP94 (hazard ratio [HR] = 2.12 [95% CI 1.23–3.04]; *P* = 0.007) (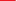) Hi and (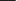) Lo. (**b**) Lung AD patients with a high FOXP3^+^ Treg count had a significantly shorter overall survival (HR = 1.73 [95% CI 1.09–2.75]; *P* = 0.016) (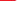) Hi and (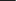) Lo.

We further examined levels of the GRP94 protein in 80 lung AD tissue samples by IHC. The identified clinicopathological characteristics related to the expression of GRP94 in lung AD are shown in Table 2. The presence of GRP94 was significantly correlated with tumor differentiation (*P* = 0.027) and disease stage (*P* = 0.022). A Kaplan‐Meier analysis was used to evaluate the survival rate of patients with lung AD. Samples were divided into two groups based on whether they had low (*n* = 28) or high (*n* = 52) GRP94 protein levels. Lung AD patients with high levels of GRP94 had a significantly shorter overall survival than those with low levels of GRP94 (hazcfard ratio [HR] = 2.12 [95% CI 1.23–3.04]; *P* = 0.007) (Fig 4a).

**Table 2:** Ggp94 expression in 80 lung AD patients

		Grp94 expression		
Variables	Cases, *n* = 80	Low, *n* = 28 (%)	High, *n* = 52 (%)	*P*‐value
Age (years)				0.761
> = 60	41	15 (53.6)	26 (50)	
<60	39	13 (46.4)	26 (50)	
Gender				0.147
Male	32	12 (42.9)	20 (38.6)	
Female	48	16 (57.1)	32 (61.5)	
Differentiation				**0.027**
Low	27	5 (21.4)	22 (40.4)	
Moderate/High	53	23 (78.6)	30 (59.6)	
pT stage				0.279
T1/T2	60	19 (67.9)	41 (78.8)	
T3/T4	20	9 (32.1)	11 (21.2)	
pN stage				0.639
N0	40	15 (53.6)	25 (48.1)	
N1/N2	40	13 (46.4)	27 (51.9)	
Tumor stage				**0.022**
I	32	16 (57.1)	16 (30.8)	
II–III	48	12 (42.9)	36 (69.2)	

### High levels of GRP94 are associated with Treg infiltration

In order to investigate the interaction between GRP94 and Treg infiltration in a lung AD microenvironment, we also examined the expression level of FOXP3. We found that FOXP3^+^ Tregs infiltrated into the tumor microenvironment (Fig 3c). We found that levels of GRP94 were significantly associated with FOXP3^+^ Treg cell counting (Table 3) and that lung AD patients with a high FOXP3^+^ Treg count had a significantly shorter overall survival (HR = 1.73 [95% CI 1.09–2.75]; *P* = 0.016) (Fig 4b).

**Table 3:** Grp94 expression interaction with Foxp3+ Tregs

	Grp94 expression		
Foxp3+ Tregs	Low, *n* = 28	High, *n* = 52	*P*‐value
High	10 (35.7)	37 (71.2)	**0.002**
Low	18 (64.3)	15 (28.8)	

### GRP94 promotes cell proliferation and inhibits cell apoptosis in A549 cells

**Figure 5:**
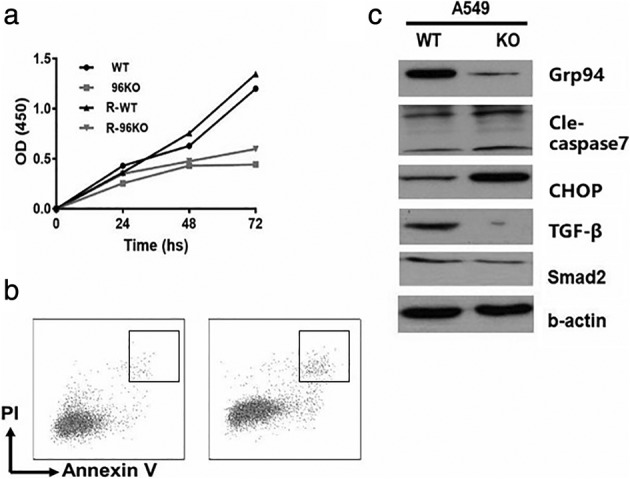
GRP94 promotes cell proliferation and inhibits cell apoptosis in A549 cells. (**a**) A CCK‐8 method was used to observe the effect of GRP94 on the proliferation of A549 cells. GRP94 gene knockout significantly inhibited A549 cell proliferation (*P* < 0.05). (**b**) The effect of GRP94 on lung AD cell apoptosis was evaluated using flow cytometric analysis. GRP94 knockdown had a significant effect on the rate of cell apoptosis (*P* < 0.01). (**c**) Western blotting was used to detect apoptosis‐related proteins. GRP94 knockout significantly increased the level of the apoptosis‐inducing proteins, CHOP and caspase‐7. The depletion of GRP94 significantly reduced levels of TGF‐β and SMAD2.

In order to determine the proliferative function of GRP94 in lung AD, we used RNA interference to inhibit GRP94 translation. Compared with the scrambled siRNA group, A549 cells transfected with a GRP94 siRNA showed significant downregulation of GRP94, which was confirmed by western blotting (Fig 5c). A CCK‐8 method was used to observe the effect of GRP94 on the proliferation of A549 cells. GRP94 gene knockout significantly inhibited A549 cell proliferation (*P* < 0.05) (Fig 5a).

The effect of GRP94 on lung AD cell apoptosis was evaluated using flow cytometric analysis. GRP94 knockdown had a significant effect on the rate of cell apoptosis (*P* < 0.01) (Fig 5b).

To further study the effect of an *HSP90B1* knockout on apoptosis, western blotting was used to detect apoptosis‐related proteins. We found that an *HSP90B1* knockout significantly increased the level of the apoptosis‐inducing proteins, CHOP and caspase‐7 (Fig 5c). These results demonstrate that loss of GRP94 inhibits cell proliferation and increases the rate of cell apoptosis.

### GRP94 may affect cell behavior via the TGF‐β signaling pathway

**Figure 6:**
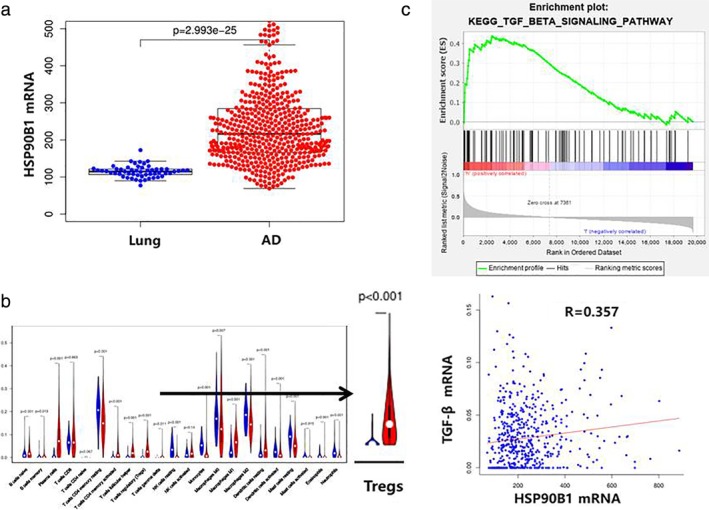
Bioinformatics analysis using the gene set enrichment analysis (GSEA) and The Cancer Genome Atlas TCGA databases. (**a**) HSP90B1 mRNA expression was significantly elevated in lung AD samples compared to normal lung tissue. (**b**) The level of Treg infiltration was also higher in lung AD tissue than in normal lung tissue. (**c**) A GSEA of *HSP90B1* revealed that the TGF‐β signaling pathway was enriched in lung AD tissue. The TGF‐β mRNA level was positively correlated with *HSP90B1* expression (R = 0.357, *P* < 0.0001).

In order to further explore the mechanism by which GRP94 promotes the proliferation of lung AD cells, we evaluated whether the absence of GRP94 resulted in the inhibition of the TGF‐β signaling pathway. Western blotting showed that depletion of GRP94 significantly reduced levels of TGF‐β and SMAD2 (Fig 5c). Furthermore, we conducted bioinformatics analyses using the Gene Set Enrichment Analysis (GSEA) and The Cancer Genome Atlas TCGA databases. We found that HSP90B1 mRNA expression was significantly elevated in lung AD samples compared to normal lung tissue (Fig 6a). The level of Treg infiltration was also higher in lung AD tissue than in normal lung tissue (Fig 6b). A GSEA of *HSP90B1* revealed that the TGF‐β signaling pathway was enriched in lung AD tissue (Fig 6c). The TGF‐β mRNA level was positively correlated with *HSP90B1* expression (R = 0.357, *P* < 0.0001).

## Discussion

Cigarette smoke is the most relevant environmental risk factor associated with the development of chronic obstructive pulmonary disease (COPD) and lung malignancies. It is well established that cigarette smoke induces ER stress, which also occurs in COPD subjects, as demonstrated by the expression of ER stress markers in fully differentiated normal human bronchial epithelial cells.^20^ Chronic ER stress resulting from exposure to cigarette smoke or another etiological agent may play a key role in the development or progression of lung cancer.^21^ GRP94 is a master ER chaperone that links protein quality control to ER stress, and inflammation and has been found to promote cancer in multiple myeloma,^13^ colitis‐associated colon tumors,^14^, ^15^ and liver cancer.^16^, ^17^, ^18^ Furthermore, Wang *et al*.^22^ revealed that GRP94 was overexpressed in lung cancer at both the mRNA and protein level and that this correlated with disease progression and poor differentiation.

In this study, we pursued a comprehensive molecular profiling of GRP94. We found that GRP94 was upregulated in human lung AD compared with normal lung tissue and that elevated GRP94 levels were associated with an aggressive phenotype and a poor clinical outcome. Increased GRP94 also correlated with poor tumor differentiation and an advanced disease stage. GRP94 has consistently been identified as a prognostic factor for overall survival and progression‐free survival in lung AD patients, but not in lung SCC patients.

GRP94 plays a key role in regulating the balance between cancer cell activity and apoptosis under many stress conditions. We have confirmed that GRP94 expression is not upregulated in lymph node metastases compared with primary lung AD tissue (data not shown). In this article, we next explored the effect of GRP94 on proliferation and apoptosis in lung AD cells. Our results showed that after depletion of GRP94 in A549 cells, their proliferative ability decreased whilst the rate of cell apoptosis increased, the latter of which occurs, in part, due to an increased level of the apoptosis‐inducing proteins, CHOP and caspase‐7.

The biological process of inflammation depends on the recruitment of various types of immune cells. CD4^+^ Tregs have been revealed as a key player in many inflammatory diseases, including cancer. Tregs can become a positive factor in cancer progression by suppressing antitumor effector cells.^23^ A previous study of NSCLC showed that increased number of FOXP3^+^ lymphocytes in tumors were associated with a reduced rate of relapse‐free survival.^24^ In this study, we have confirmed that increased levels of GRP94 are significantly positively correlated with FOXP3^+^ Treg infiltration into lung AD tissues. Both GRP94 and Tregs have consistently been identified as prognostic factors of overall survival in lung AD, suggesting that both may play critical roles in lung cancer development and disease progression.

Mouse models of Lewis lung carcinoma have shown that Tregs inhibit natural killer (NK) cell‐mediated cytotoxicity in a TGF‐β‐dependent manner, and Treg depletion can enhance the anti‐tumor activity of NK cells.^22^ To further explore the mechanism by which GRP94 promotes the proliferation of lung AD cells, we evaluated the effect of GRP94 depletion on the TGF‐β signaling pathway. Our results showed that GRP94 depletion significantly decreased levels of TGF‐β and SMAD2. Furthermore, we conducted bioinformatics analyses using the GSEA and TCGA databases. We found that more Tregs infiltrated lung AD tissue than normal lung tissue. A GSEA analysis of *HSP90B1* revealed that the TGF‐β signaling pathway was enriched in lung AD tissue and there was an exact correlation between the mRNA levels of TGF‐β and HSP90B1. These results indicate that Treg immune cell infiltration may be involved in the oncogenic role of GRP94 in lung AD via promotion of the TGF‐β signaling pathway.

## Disclosure

No authors report any conflict of interest.

## Supporting information


**Appendix S1**: Supporting information
