# Supplementary figures and images for: Overexpression of molecule GRP94 favors tumor progression in lung adenocarcinoma by interaction with regulatory T cells
Source: Thorac Cancer. 2020 Jan 22;11(3):704–12. doi: 10.1111/1759-7714.13321 (PMC7049511; doi:10.1111/1759-7714.13321)

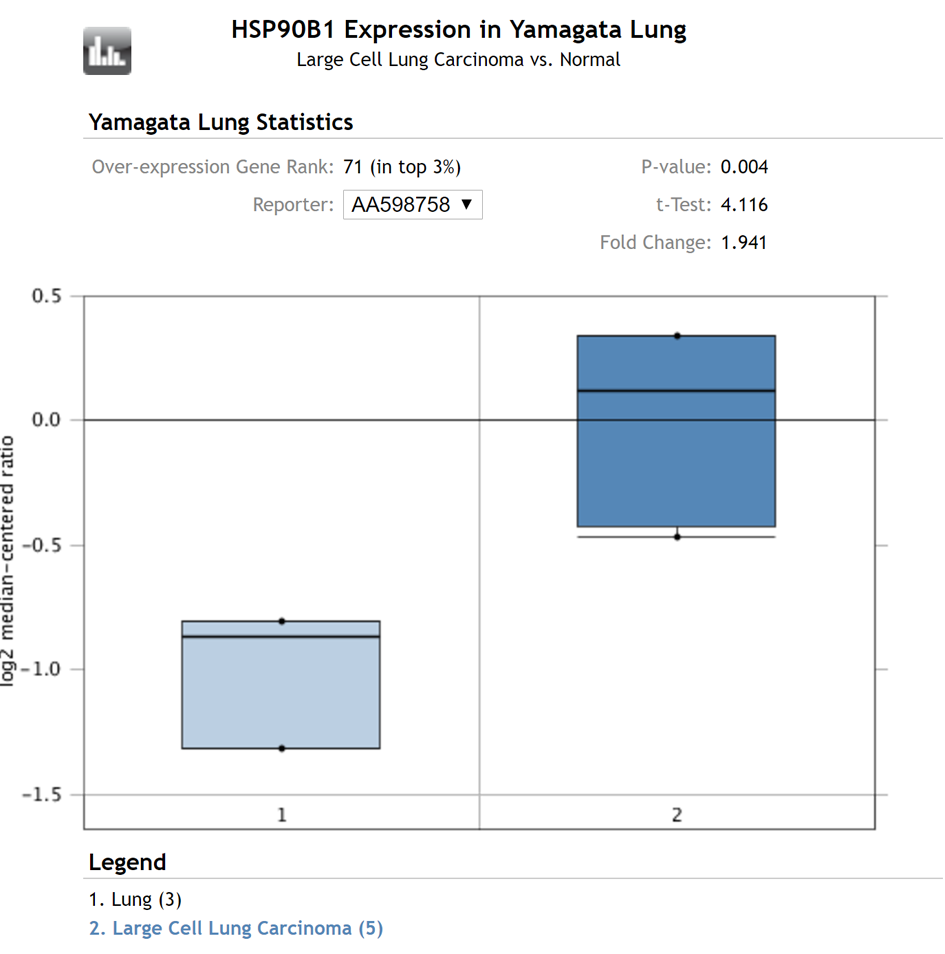

Supplement: Supplementary file 1 — Appendix S1: Supporting information [file TCA-11-704-s001.zip › Oncomine data/cancer vs normal/Lung cancer vs normal/1.tif]

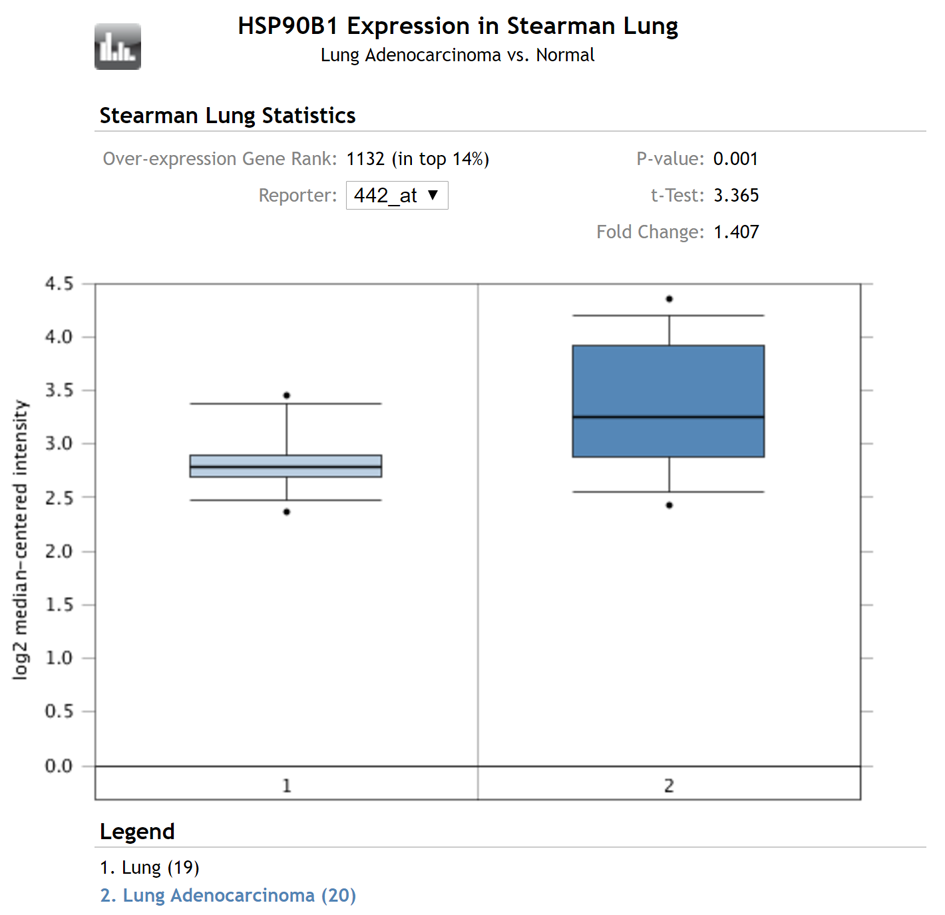

Supplement: Supplementary file 1 — Appendix S1: Supporting information [file TCA-11-704-s001.zip › Oncomine data/cancer vs normal/Lung cancer vs normal/10.tif]

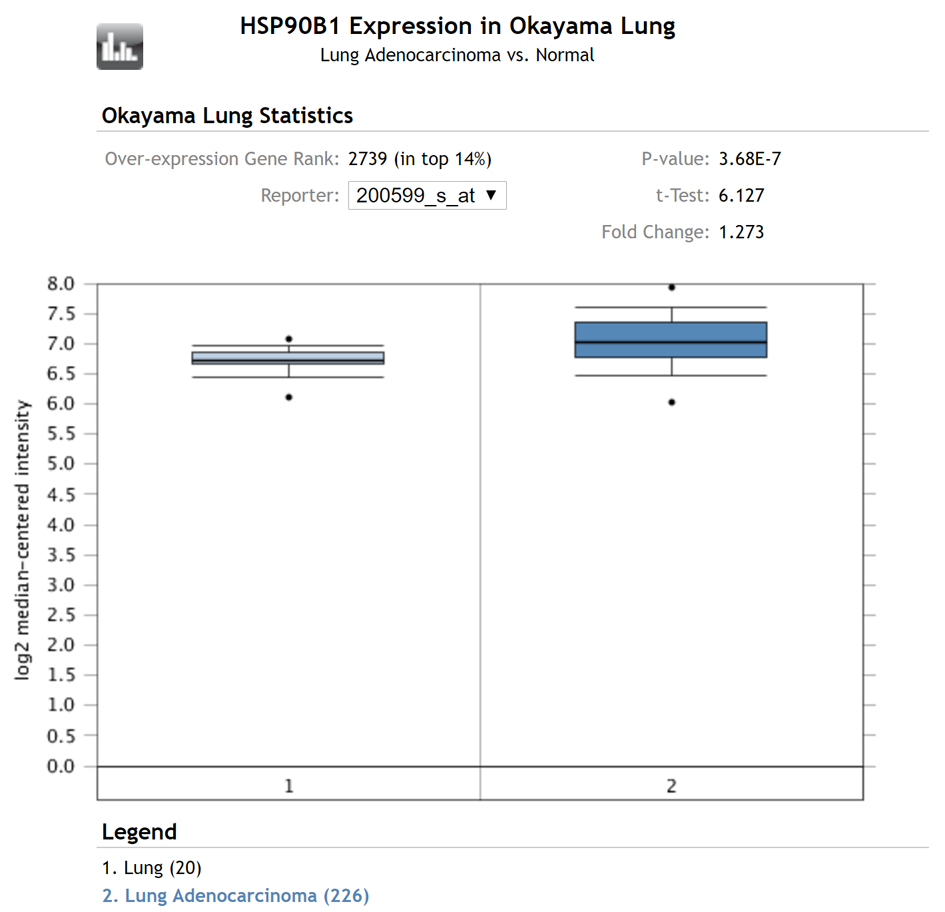

Supplement: Supplementary file 1 — Appendix S1: Supporting information [file TCA-11-704-s001.zip › Oncomine data/cancer vs normal/Lung cancer vs normal/11.tif]

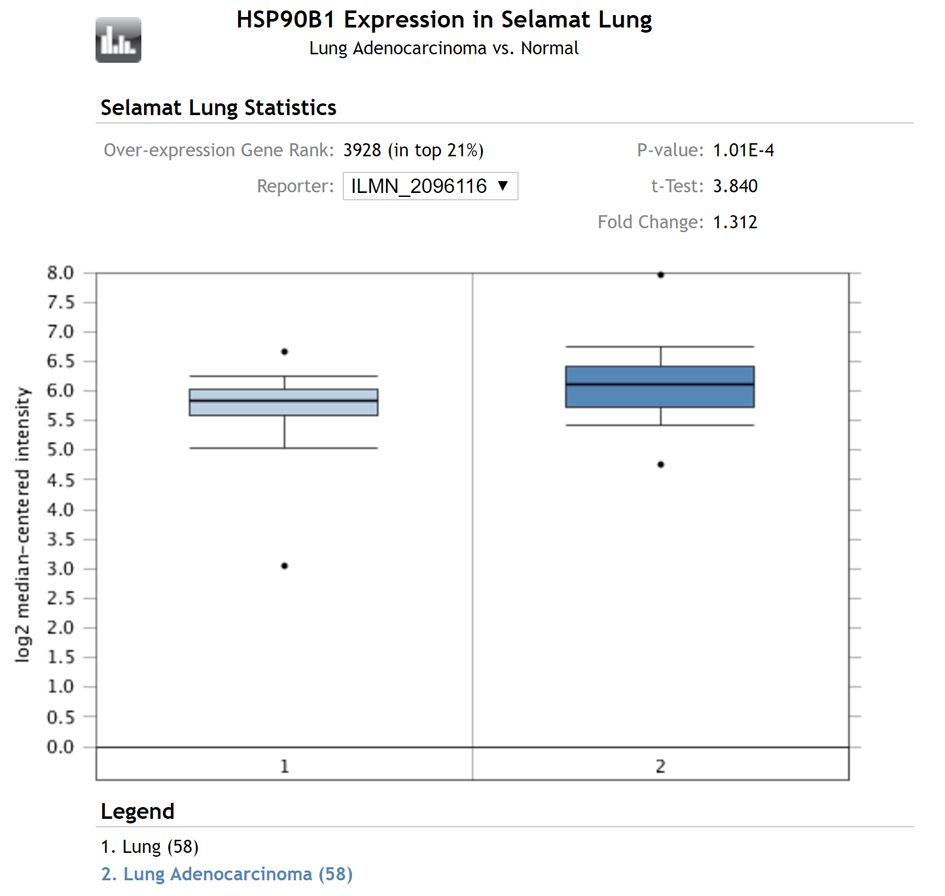

Supplement: Supplementary file 1 — Appendix S1: Supporting information [file TCA-11-704-s001.zip › Oncomine data/cancer vs normal/Lung cancer vs normal/12.tif]

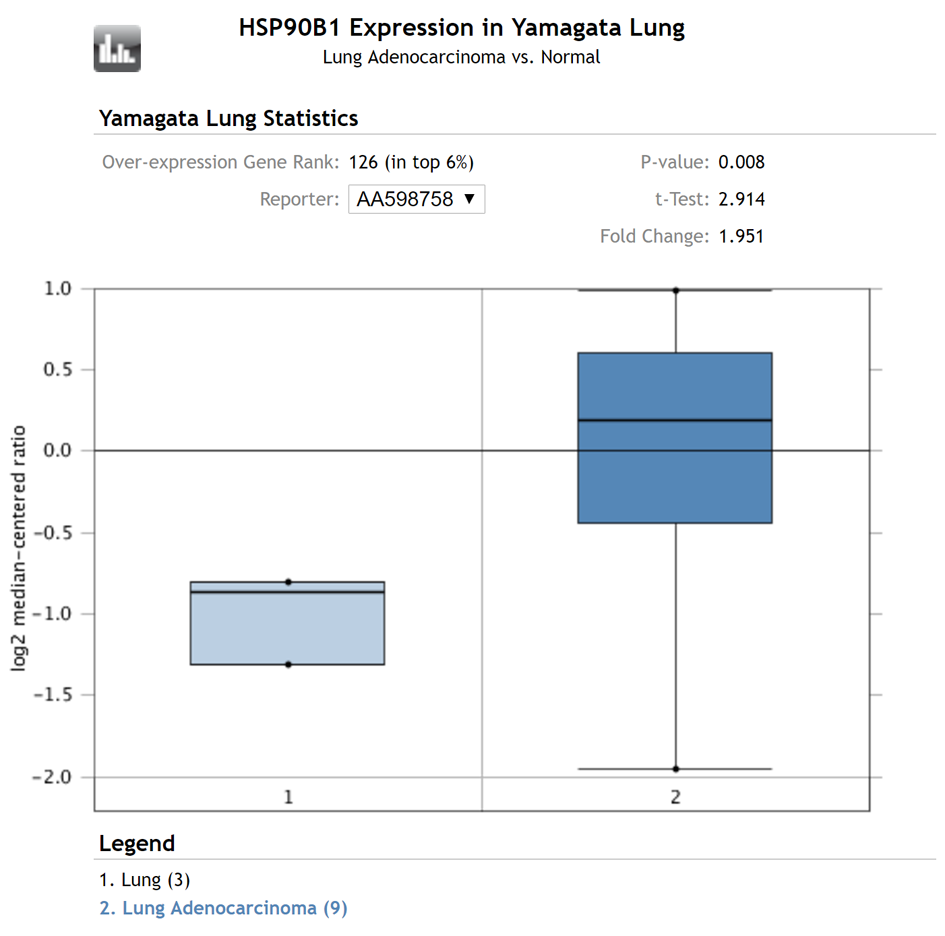

Supplement: Supplementary file 1 — Appendix S1: Supporting information [file TCA-11-704-s001.zip › Oncomine data/cancer vs normal/Lung cancer vs normal/2.tif]

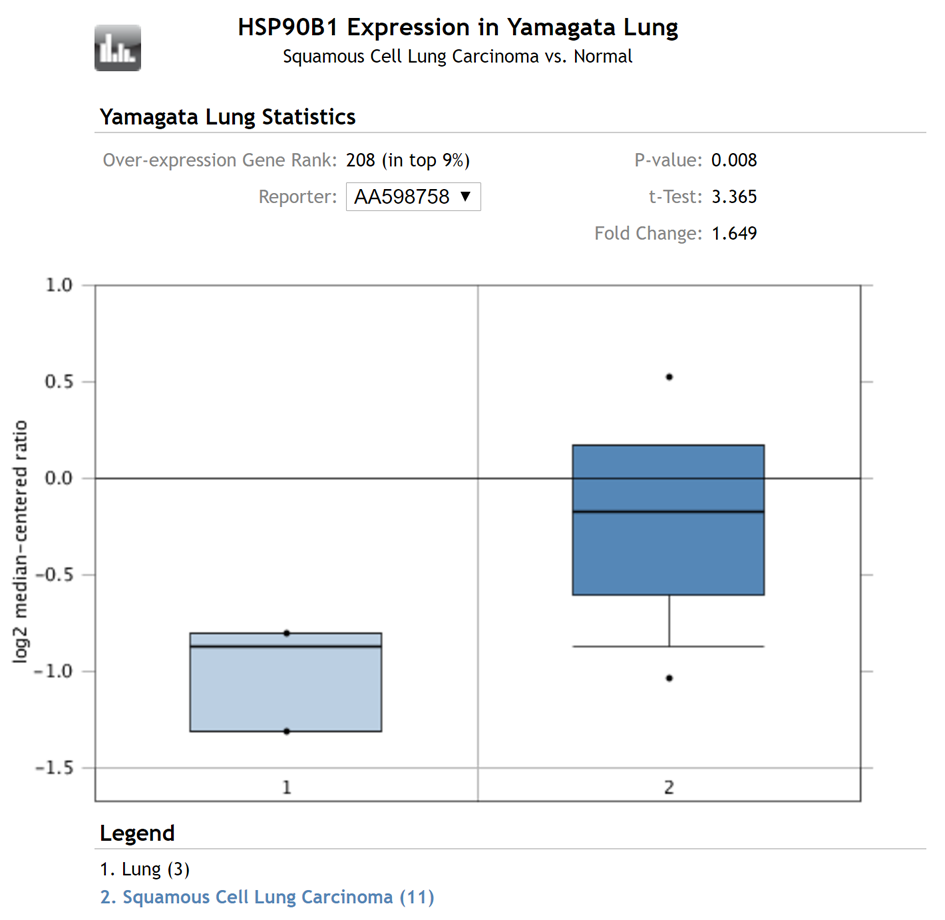

Supplement: Supplementary file 1 — Appendix S1: Supporting information [file TCA-11-704-s001.zip › Oncomine data/cancer vs normal/Lung cancer vs normal/3.tif]

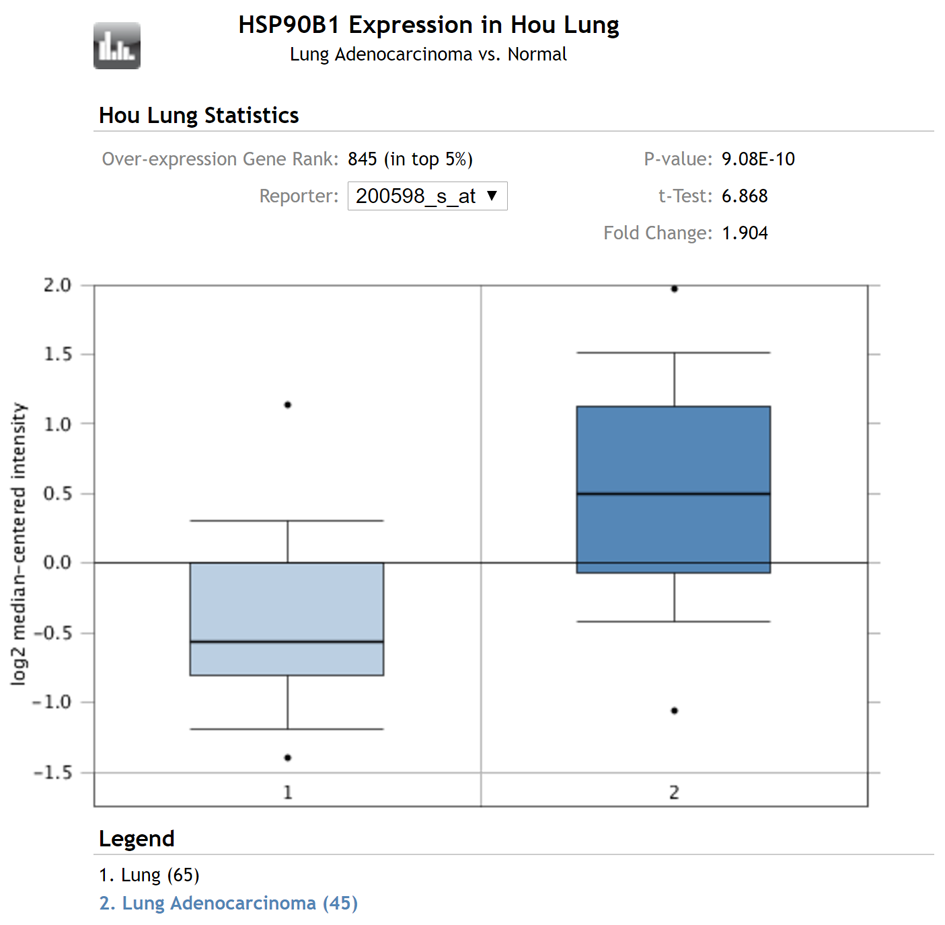

Supplement: Supplementary file 1 — Appendix S1: Supporting information [file TCA-11-704-s001.zip › Oncomine data/cancer vs normal/Lung cancer vs normal/4.tif]

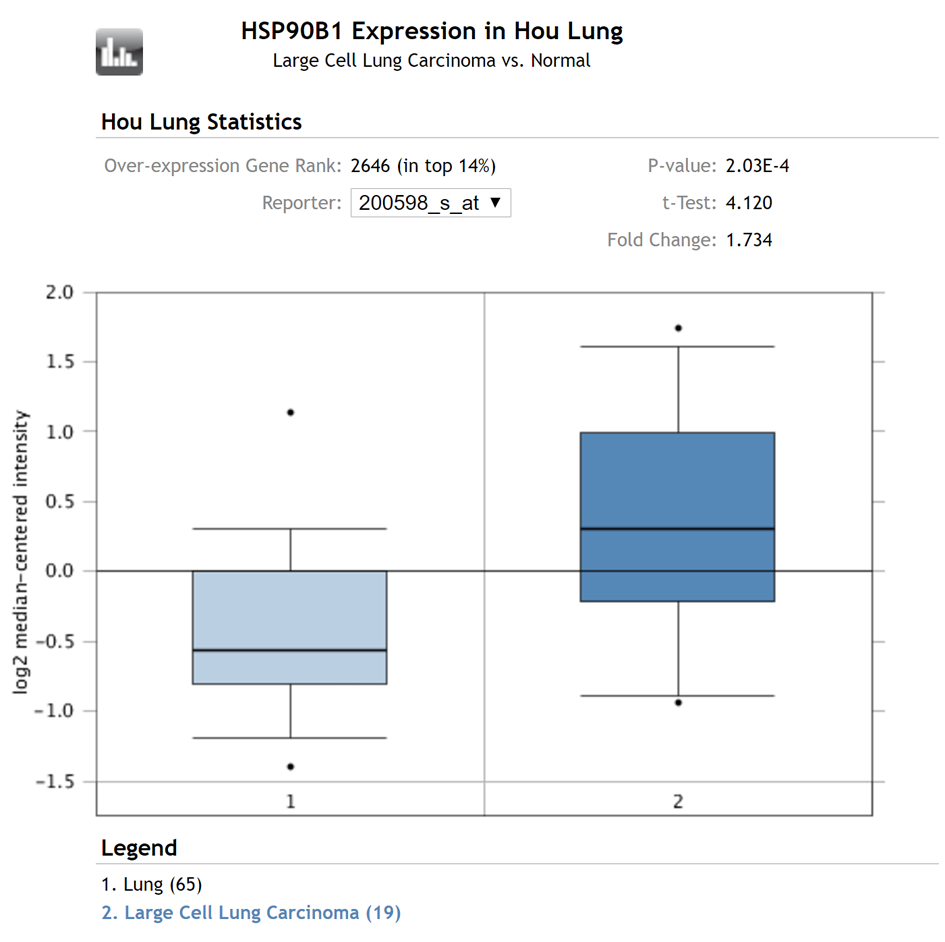

Supplement: Supplementary file 1 — Appendix S1: Supporting information [file TCA-11-704-s001.zip › Oncomine data/cancer vs normal/Lung cancer vs normal/5.tif]

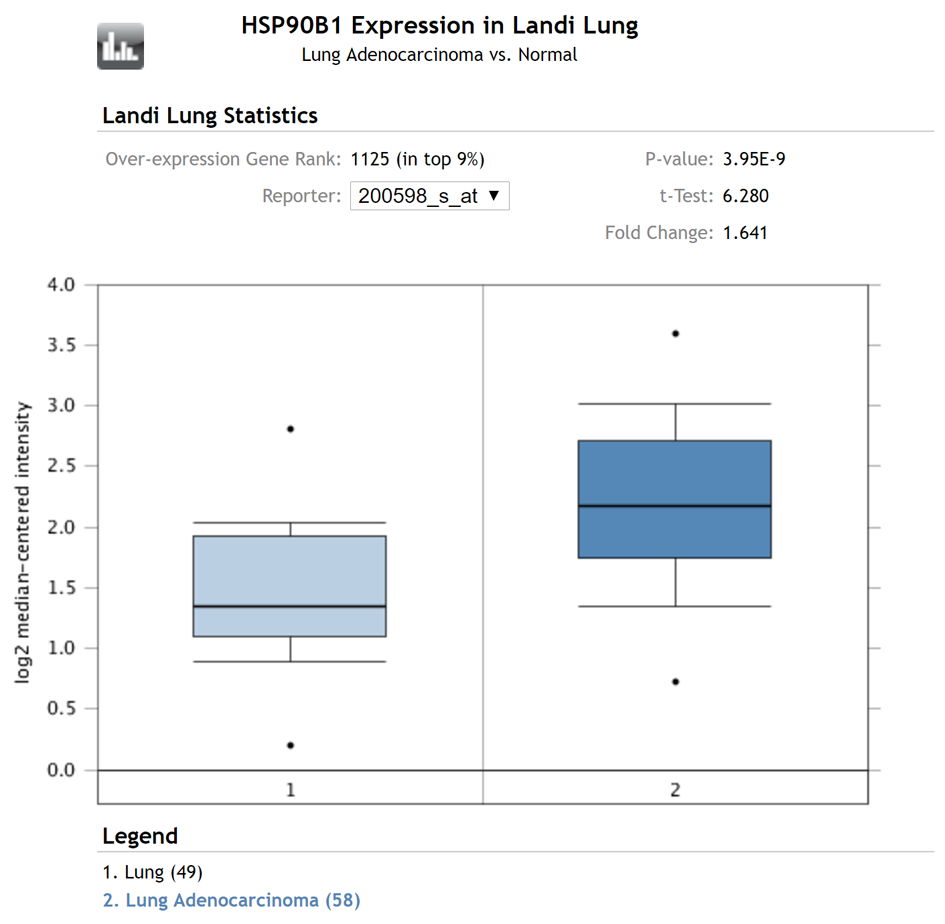

Supplement: Supplementary file 1 — Appendix S1: Supporting information [file TCA-11-704-s001.zip › Oncomine data/cancer vs normal/Lung cancer vs normal/6.tif]

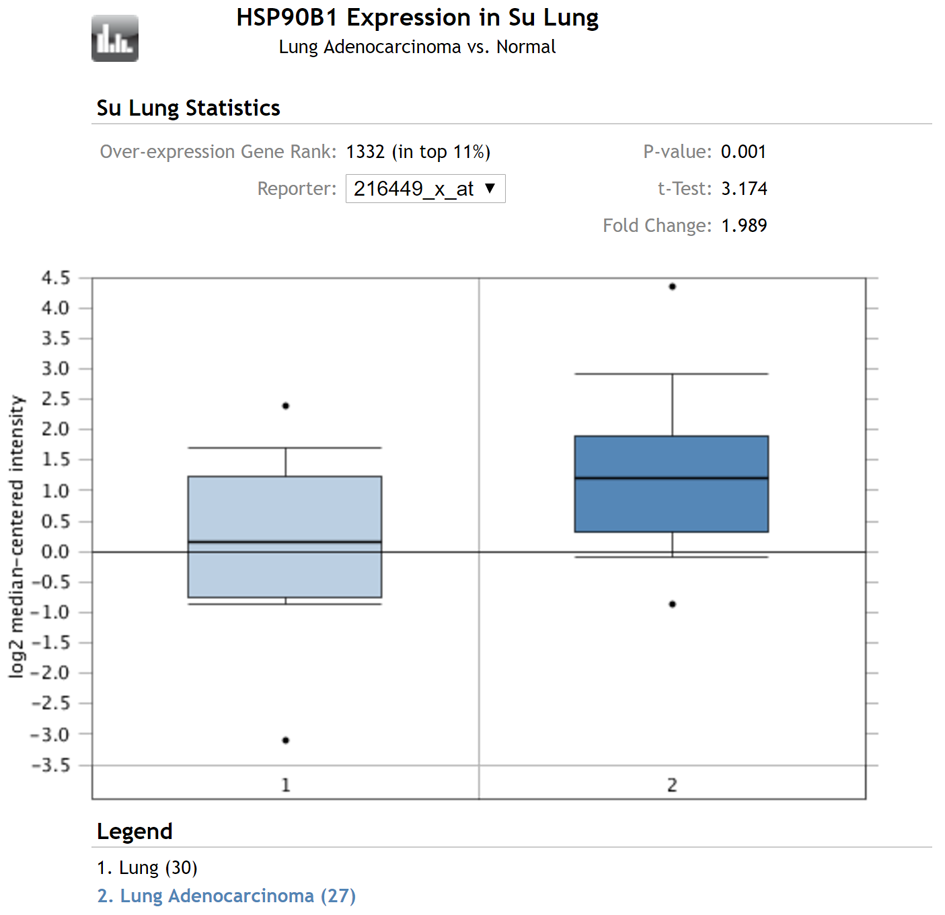

Supplement: Supplementary file 1 — Appendix S1: Supporting information [file TCA-11-704-s001.zip › Oncomine data/cancer vs normal/Lung cancer vs normal/7.tif]

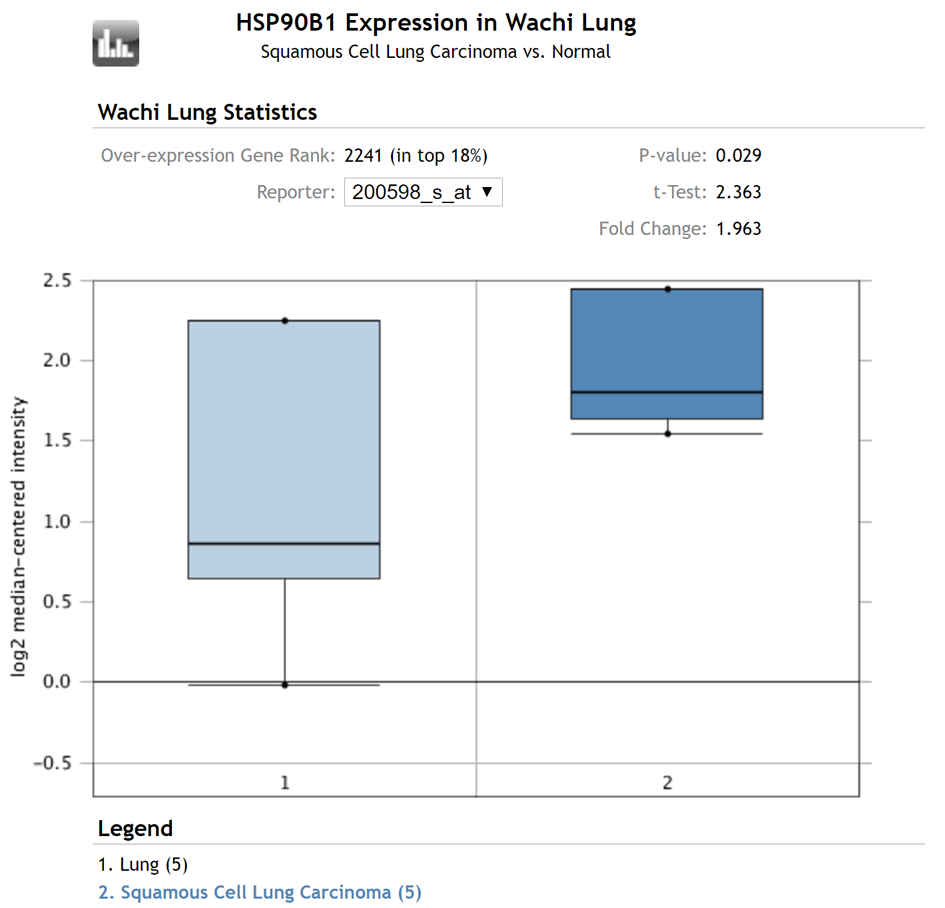

Supplement: Supplementary file 1 — Appendix S1: Supporting information [file TCA-11-704-s001.zip › Oncomine data/cancer vs normal/Lung cancer vs normal/8.tif]

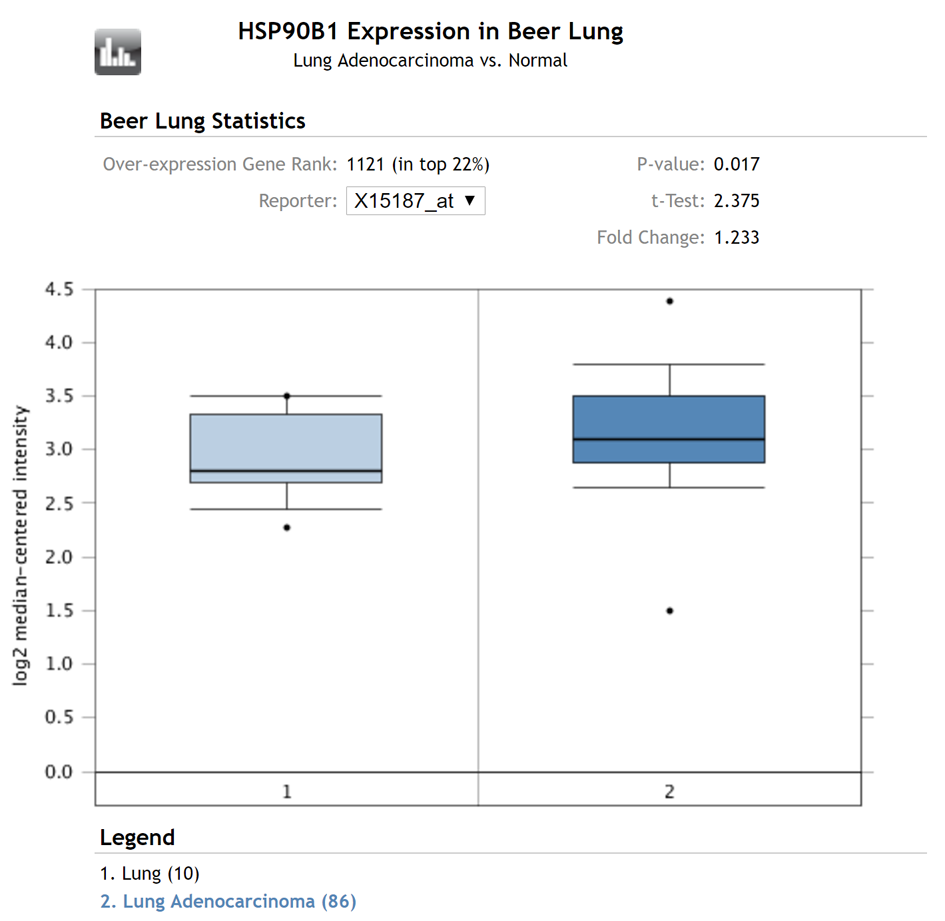

Supplement: Supplementary file 1 — Appendix S1: Supporting information [file TCA-11-704-s001.zip › Oncomine data/cancer vs normal/Lung cancer vs normal/9.tif]

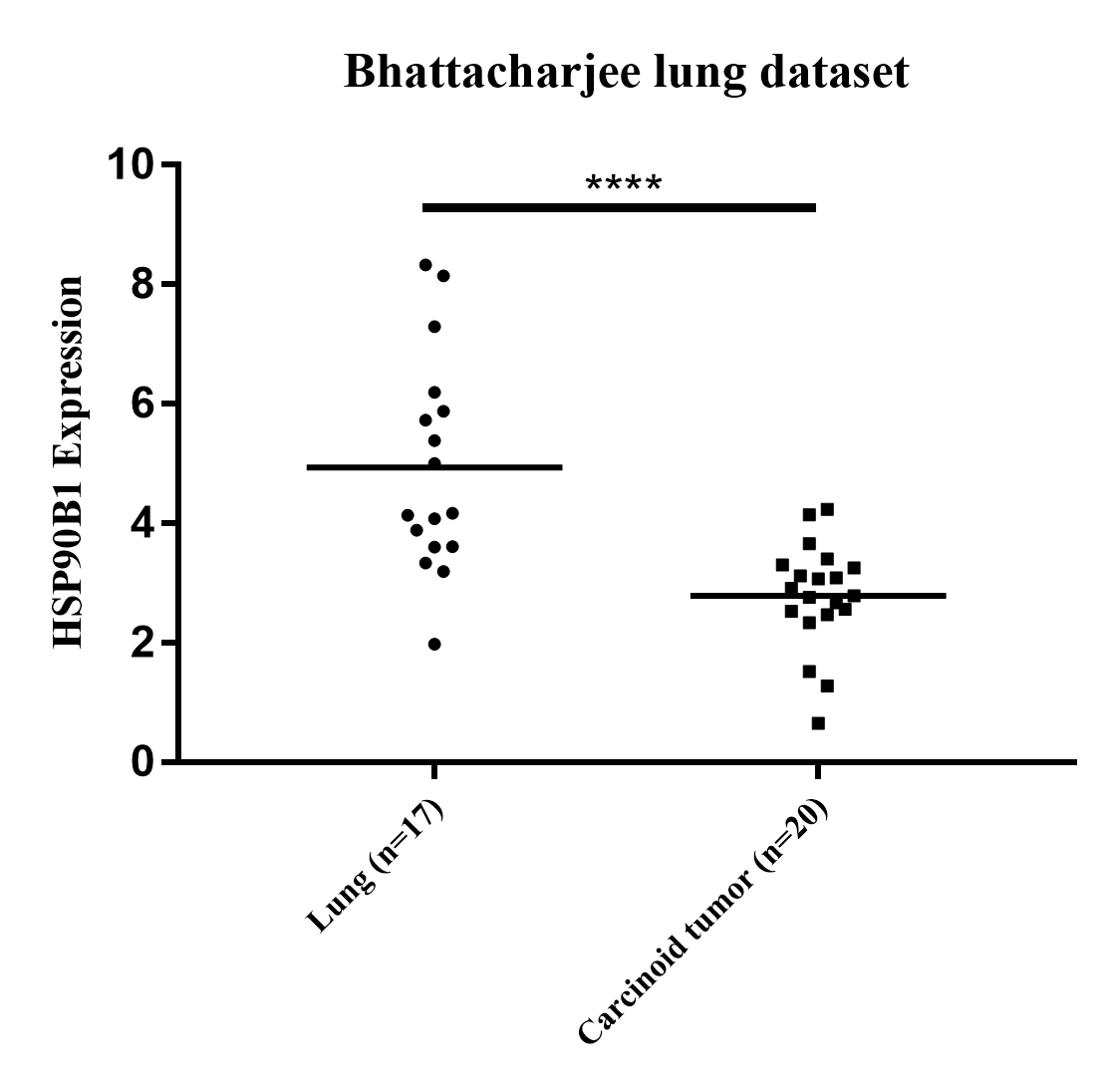

Supplement: Supplementary file 1 — Appendix S1: Supporting information [file TCA-11-704-s001.zip › Oncomine data/cancer vs normal/Lung cancer vs normal/dataset/dataset figs/Bhatacharjee lung.tif]

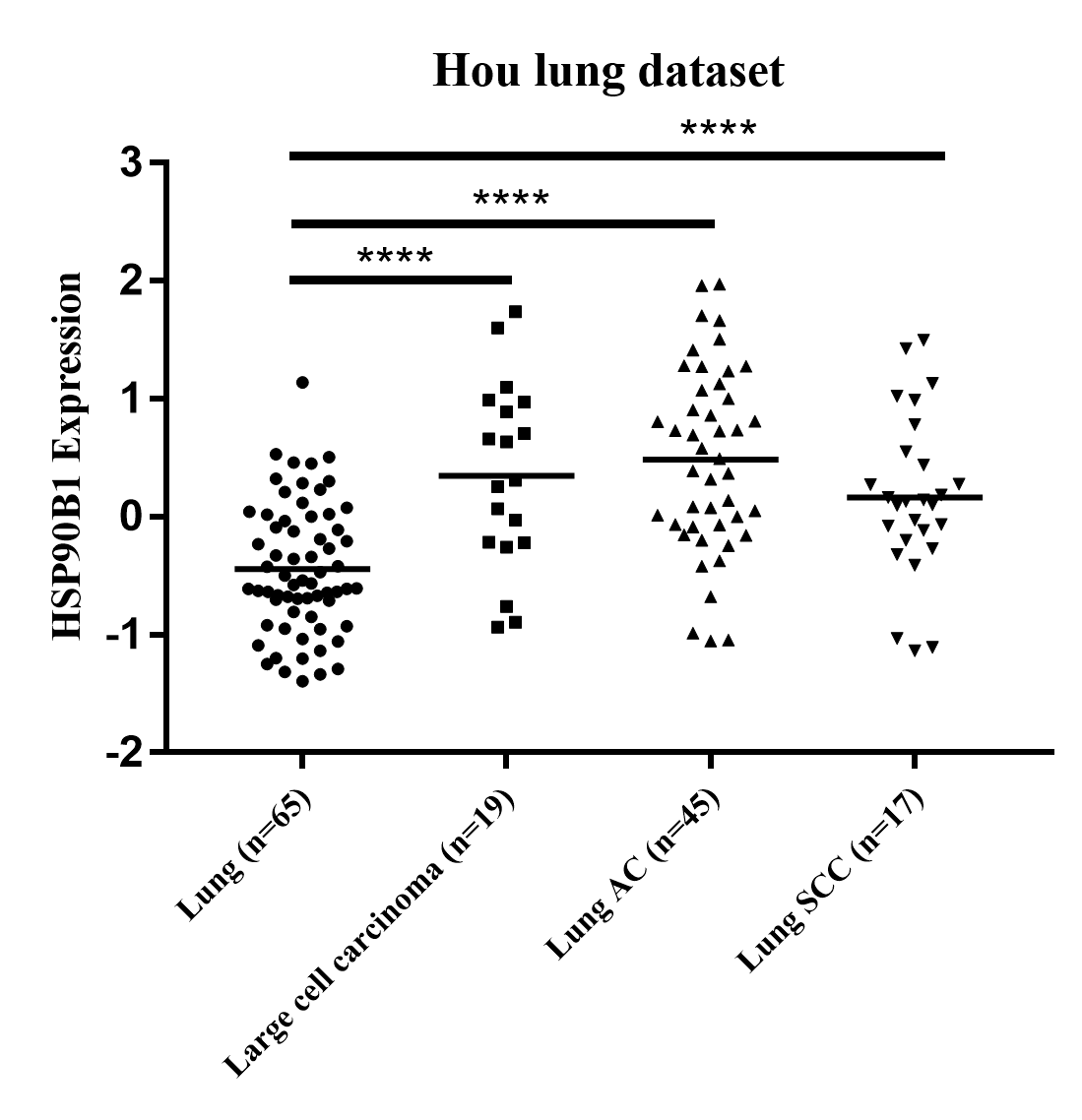

Supplement: Supplementary file 1 — Appendix S1: Supporting information [file TCA-11-704-s001.zip › Oncomine data/cancer vs normal/Lung cancer vs normal/dataset/dataset figs/Hou lung.tif]

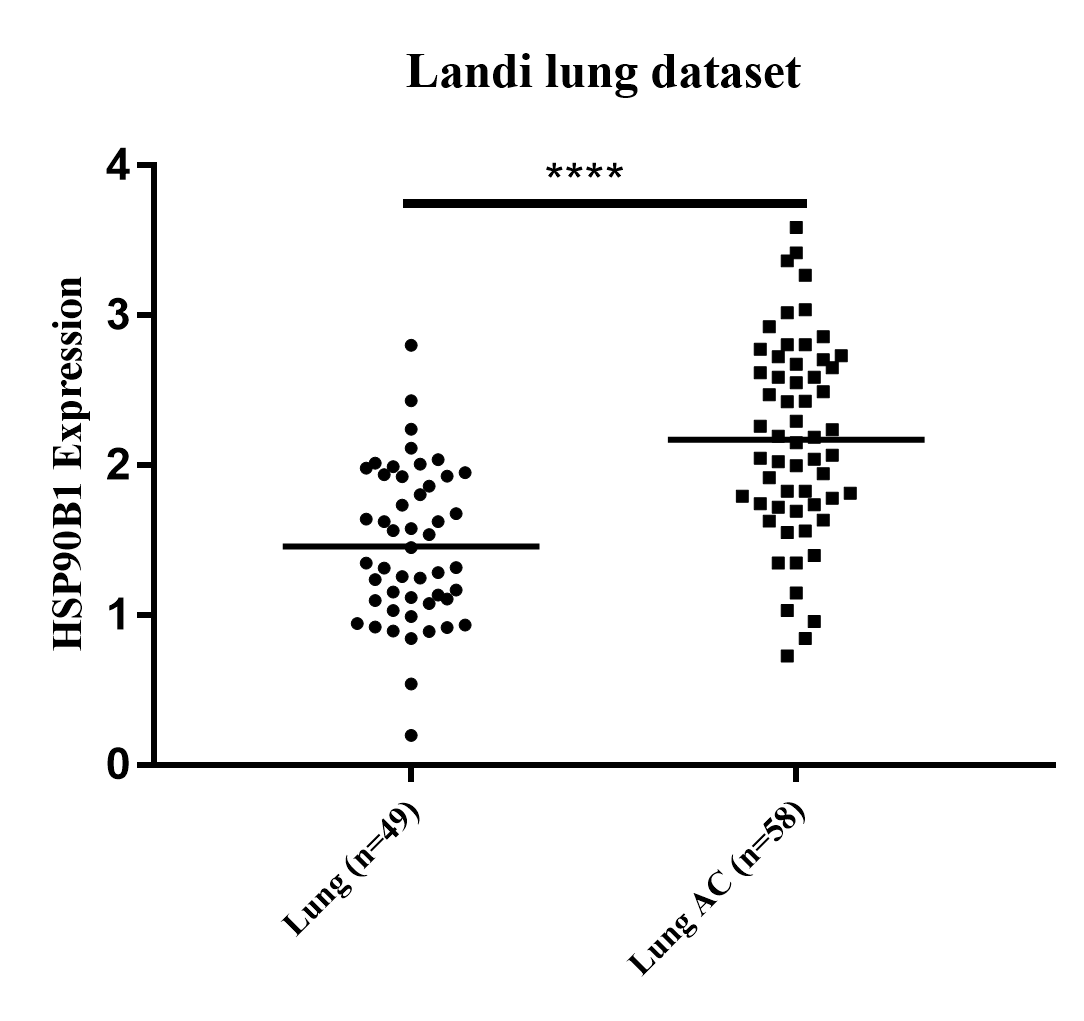

Supplement: Supplementary file 1 — Appendix S1: Supporting information [file TCA-11-704-s001.zip › Oncomine data/cancer vs normal/Lung cancer vs normal/dataset/dataset figs/Landi lung.tif]

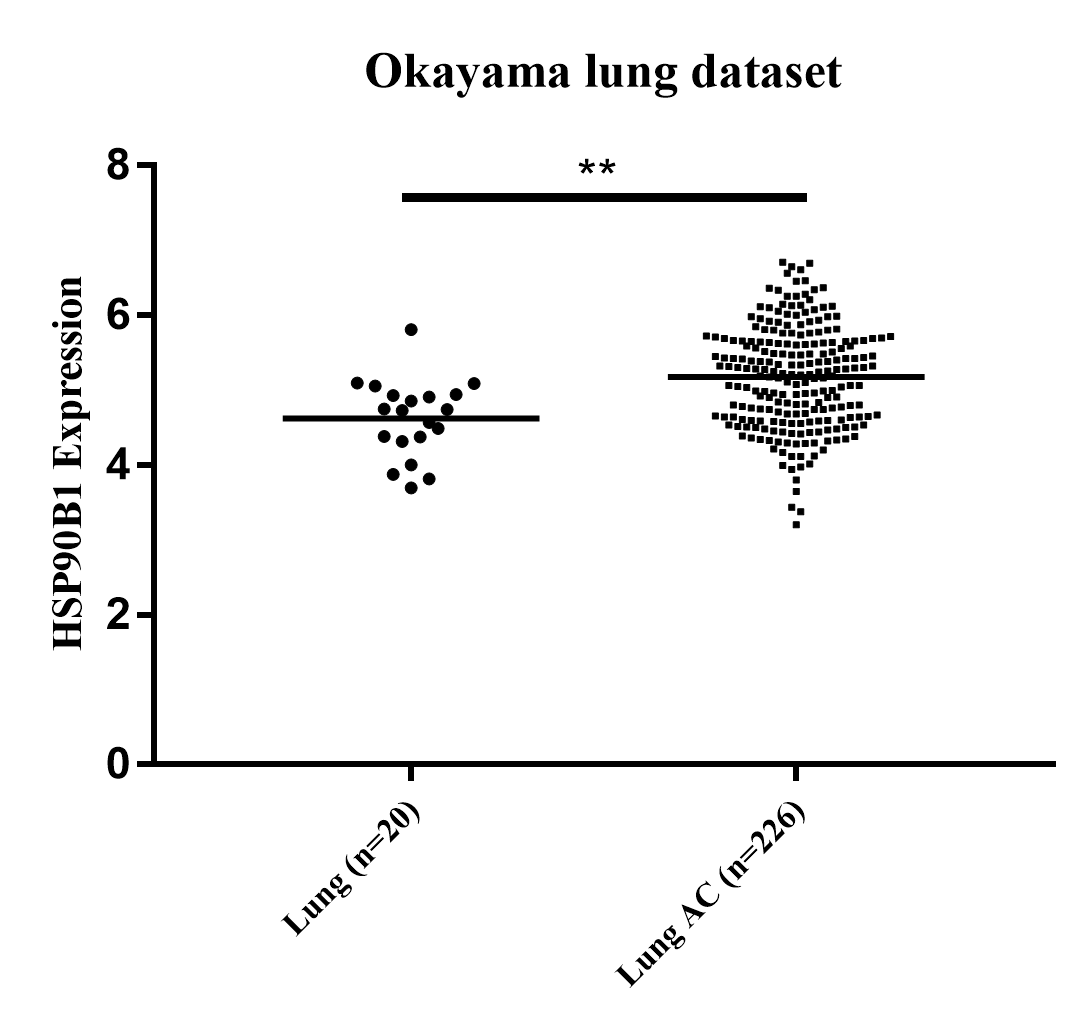

Supplement: Supplementary file 1 — Appendix S1: Supporting information [file TCA-11-704-s001.zip › Oncomine data/cancer vs normal/Lung cancer vs normal/dataset/dataset figs/OKAYAMA LUNG.tif]

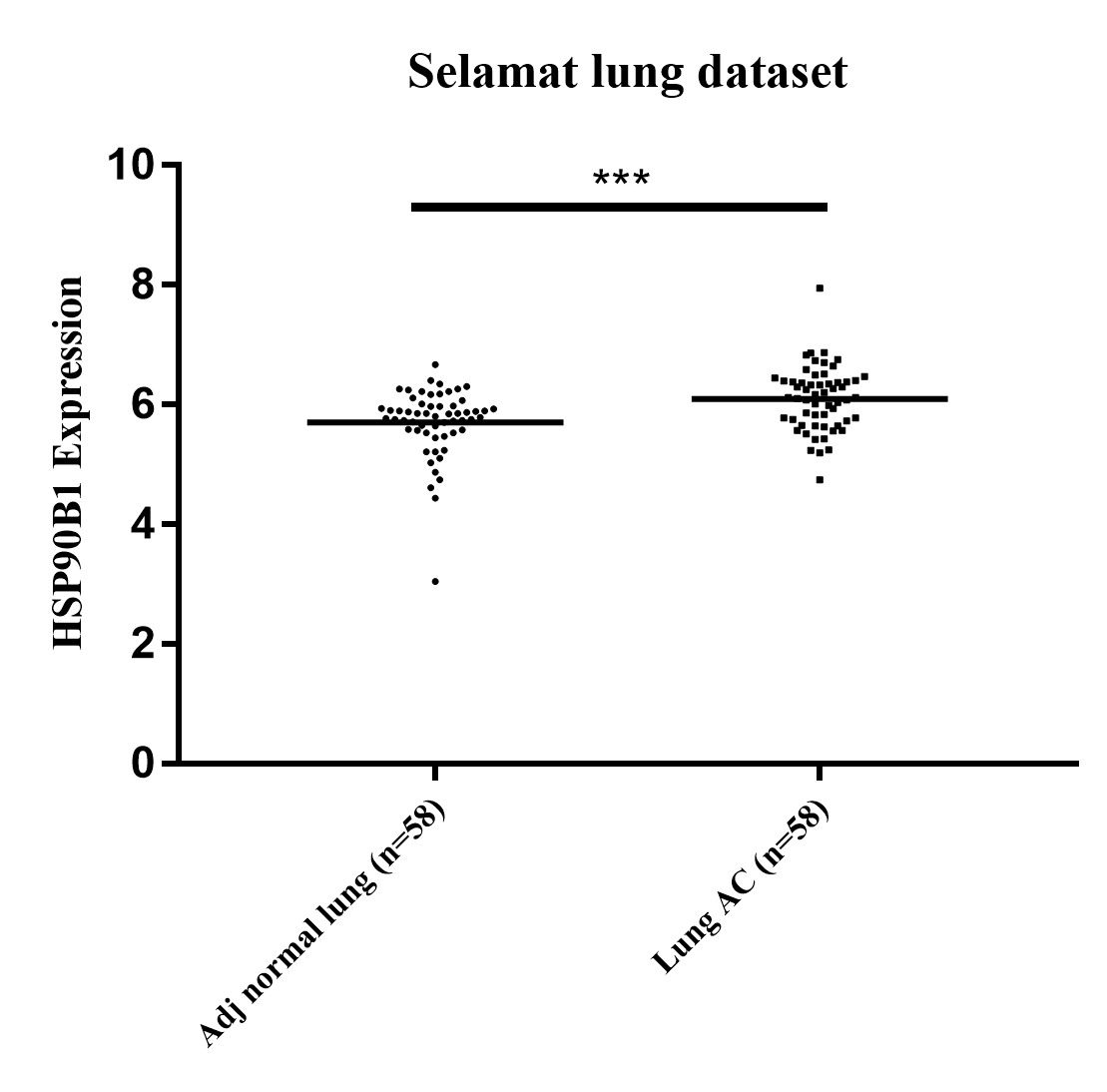

Supplement: Supplementary file 1 — Appendix S1: Supporting information [file TCA-11-704-s001.zip › Oncomine data/cancer vs normal/Lung cancer vs normal/dataset/dataset figs/Selamat lung.tif]

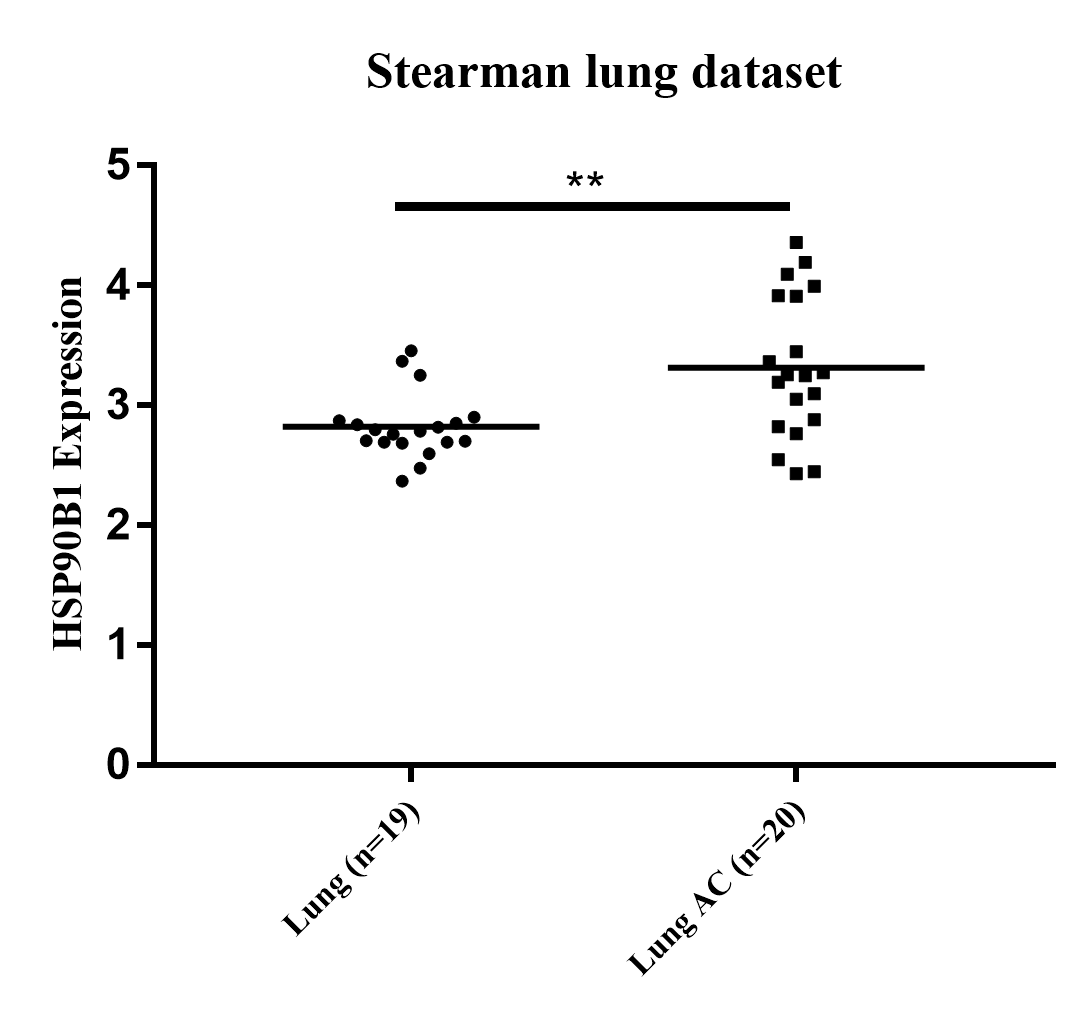

Supplement: Supplementary file 1 — Appendix S1: Supporting information [file TCA-11-704-s001.zip › Oncomine data/cancer vs normal/Lung cancer vs normal/dataset/dataset figs/Stearman lung.tif]

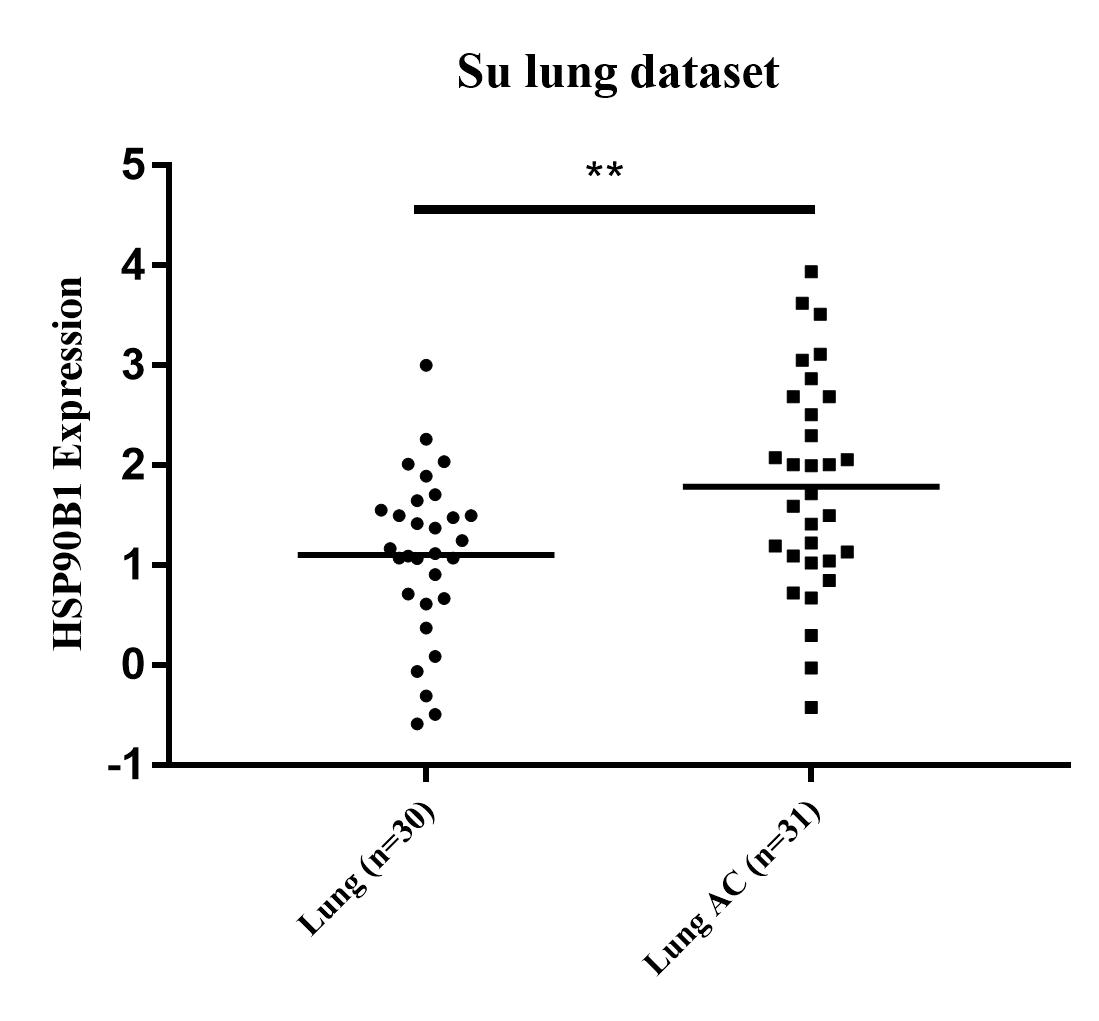

Supplement: Supplementary file 1 — Appendix S1: Supporting information [file TCA-11-704-s001.zip › Oncomine data/cancer vs normal/Lung cancer vs normal/dataset/dataset figs/Su lung.tif]

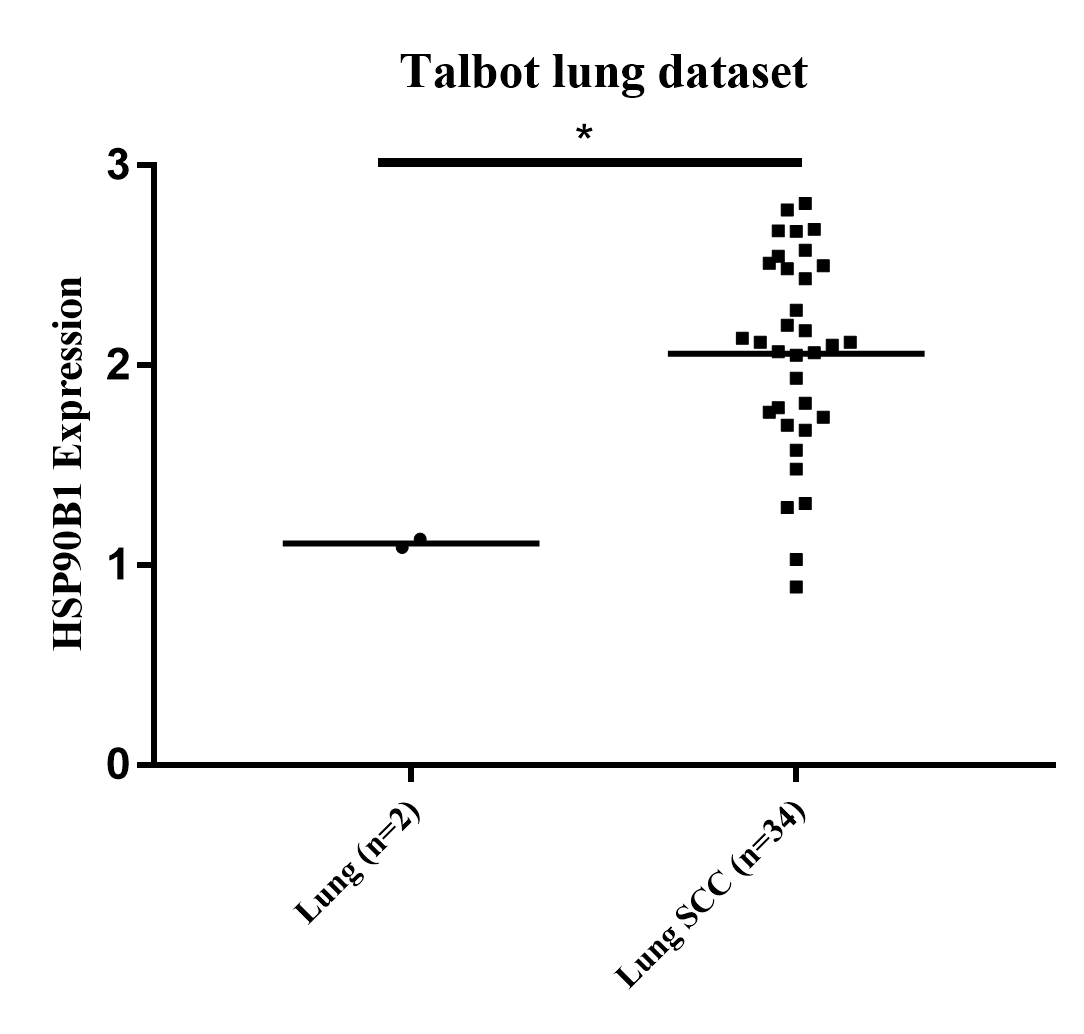

Supplement: Supplementary file 1 — Appendix S1: Supporting information [file TCA-11-704-s001.zip › Oncomine data/cancer vs normal/Lung cancer vs normal/dataset/dataset figs/Talbot lung.tif]

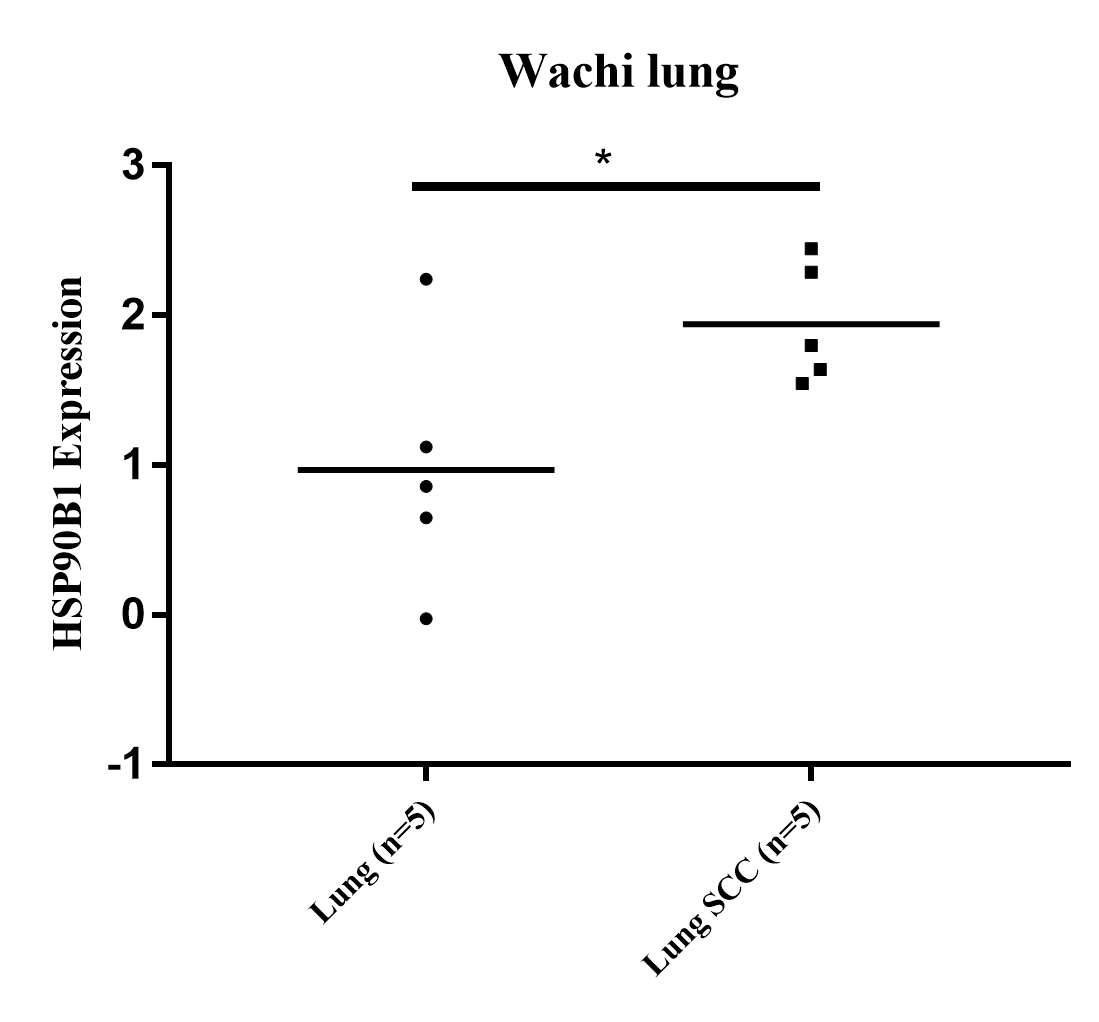

Supplement: Supplementary file 1 — Appendix S1: Supporting information [file TCA-11-704-s001.zip › Oncomine data/cancer vs normal/Lung cancer vs normal/dataset/dataset figs/Wachi lung.tif]

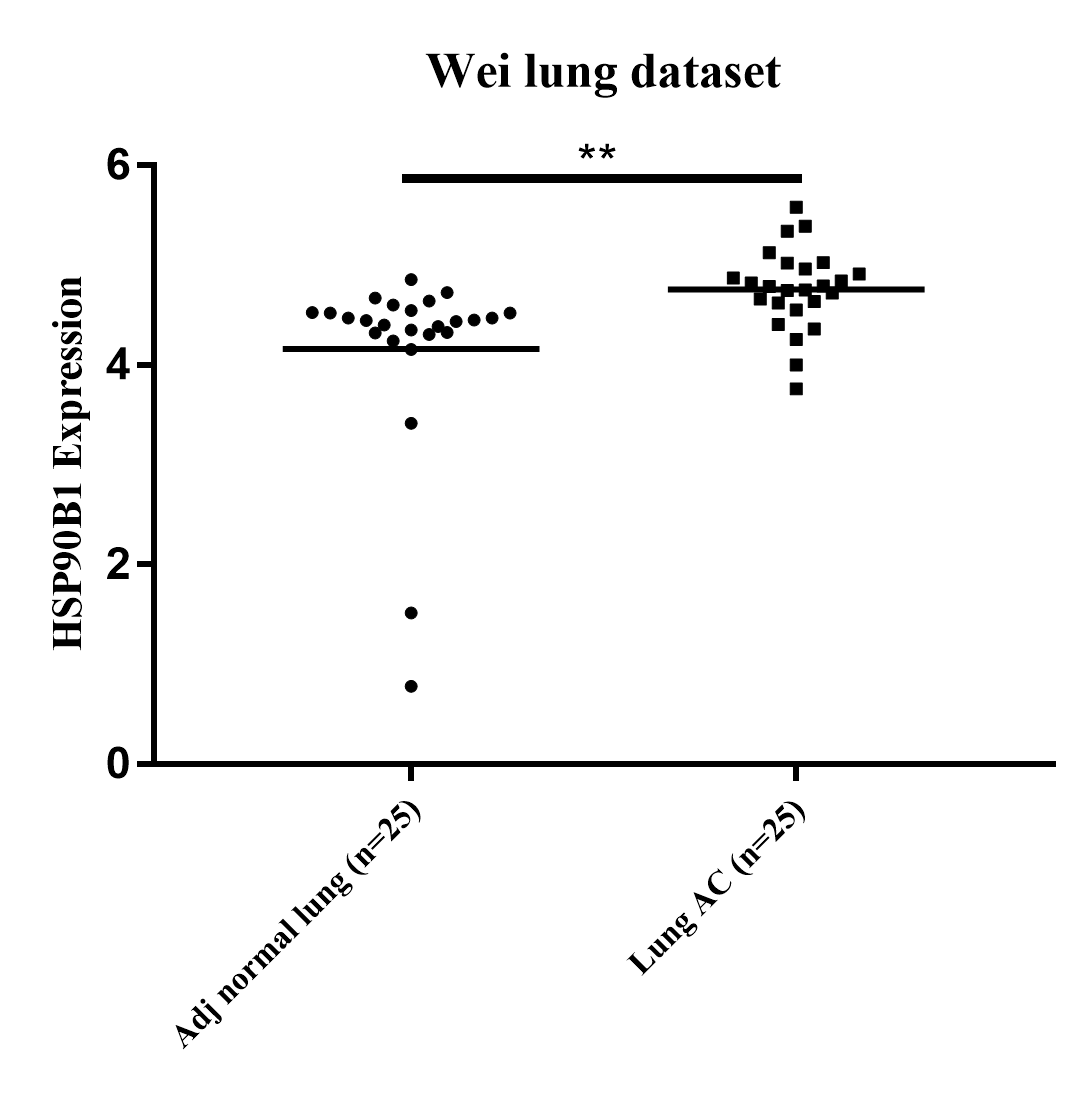

Supplement: Supplementary file 1 — Appendix S1: Supporting information [file TCA-11-704-s001.zip › Oncomine data/cancer vs normal/Lung cancer vs normal/dataset/dataset figs/Wei lung.tif]

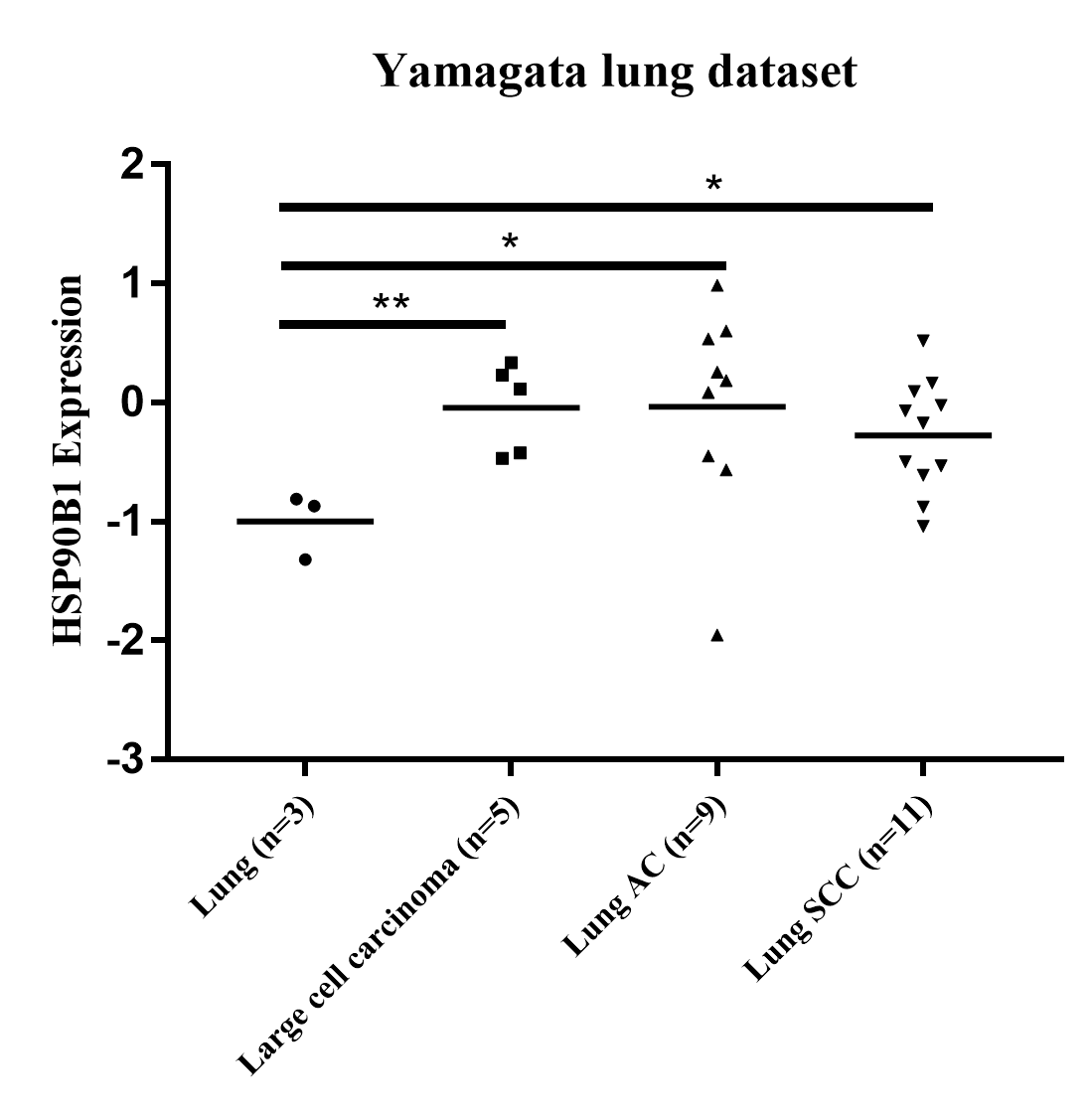

Supplement: Supplementary file 1 — Appendix S1: Supporting information [file TCA-11-704-s001.zip › Oncomine data/cancer vs normal/Lung cancer vs normal/dataset/dataset figs/Yamagata lung.tif]

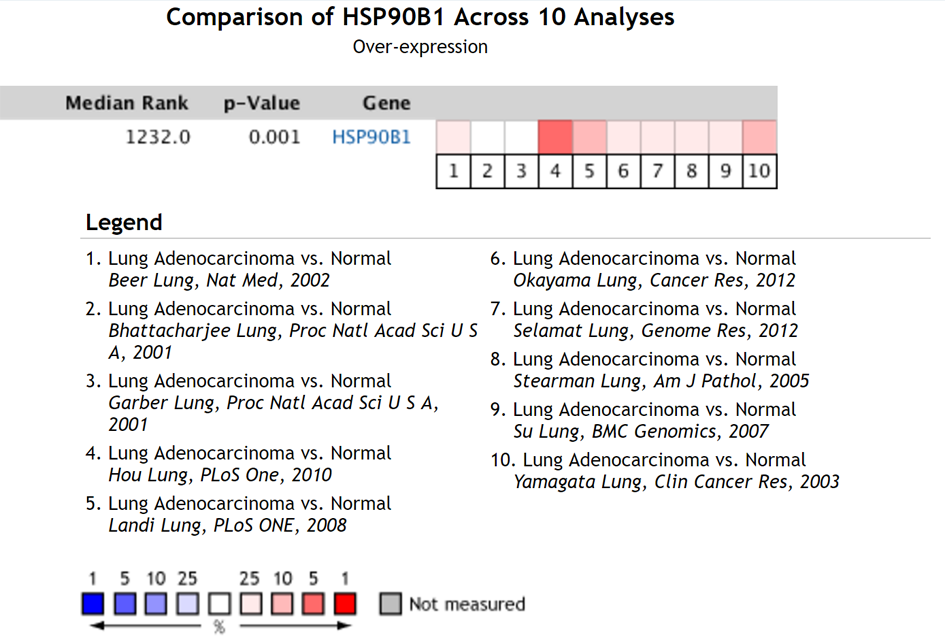

Supplement: Supplementary file 1 — Appendix S1: Supporting information [file TCA-11-704-s001.zip › Oncomine data/cancer vs normal/Lung cancer vs normal/lung aed Meta analysis.tif]

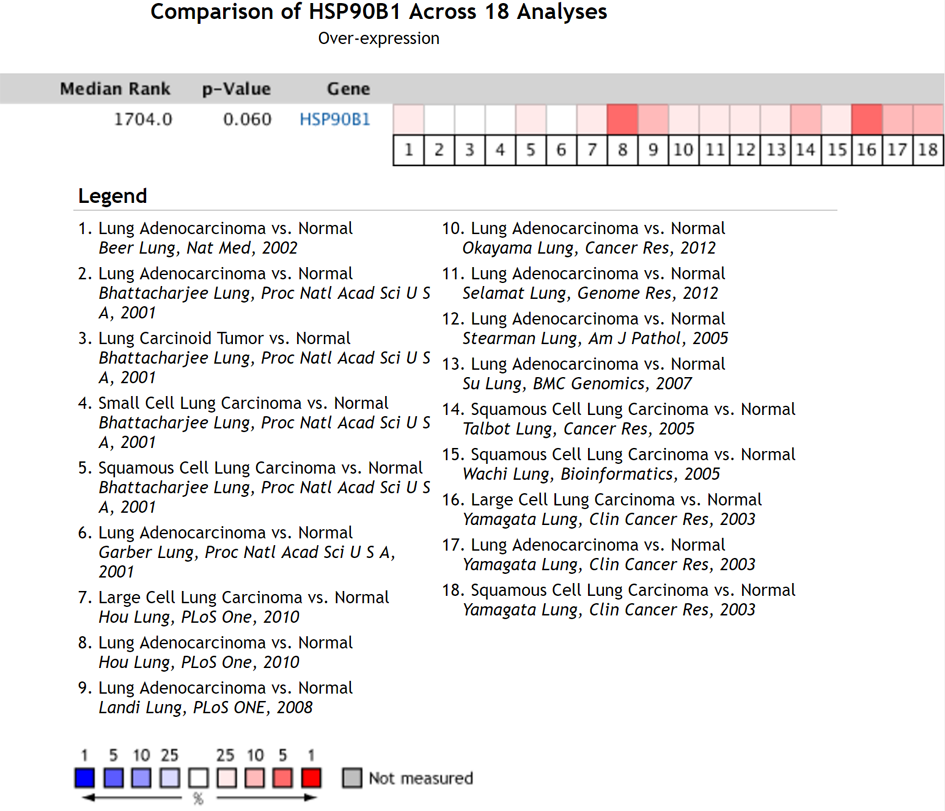

Supplement: Supplementary file 1 — Appendix S1: Supporting information [file TCA-11-704-s001.zip › Oncomine data/cancer vs normal/Lung cancer vs normal/lung cancer Meta analysis.tif]

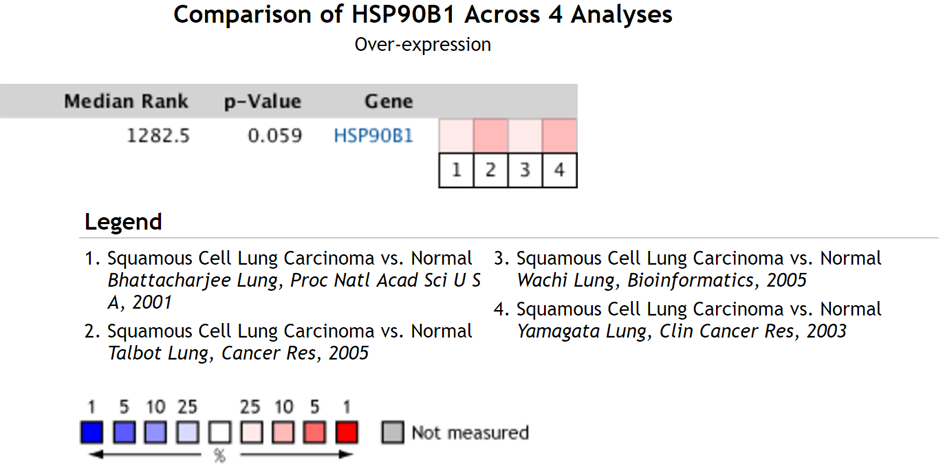

Supplement: Supplementary file 1 — Appendix S1: Supporting information [file TCA-11-704-s001.zip › Oncomine data/cancer vs normal/Lung cancer vs normal/lung SCC Meta analysis.tif]
